# Evidence for cable bacteria inhabiting deep in anoxic sediment reveals a novel ecological niche

**DOI:** 10.1186/s40793-026-00895-7

**Published:** 2026-04-15

**Authors:** Alexis Fonseca, Martijn Hermans, Francisco J. A. Nascimento, Christian Stranne, Alf Norkko, Bo G. Gustafsson, Christoph Humborg

**Affiliations:** 1 Baltic Sea Centre, Stockholm University, Stockholm, Sweden; 2 Department of Ecology, Environment and Plant Sciences, Stockholm University, Stockholm, Sweden; 3 Department of Geological Sciences, Stockholm University, Stockholm, Sweden; 4 Bolin Centre for Climate Research, Stockholm University, Stockholm, Sweden; 5 Tvärminne Zoological Station, University of Helsinki, Hanko, Finland; 6 Baltic Nest Institute, Stockholm University, Stockholm, Sweden

**Keywords:** Cable bacteria, *Candidatus* Electrothrix, Anoxic sediments, Metatranscriptomic, Sulphur bacteria, Sulphur oxidation, Nitrate reduction, Sulfammox, Novel niche, Koljö fjord

## Abstract

**Background:**

Cable bacteria are filamentous sulphide-oxidisers capable of cm-scale electron transport. They are generally considered restricted to the upper few oxic–suboxic cm of marine sediments, where they couple sulphide oxidation to oxygen or nitrate reduction. Despite their influence on redox gradients, trace metal mobility, and nutrient cycling, their presence and activity in deeper anoxic sediment layers remain unknown. The presence and activity of marine cable bacteria (*Candidatus* Electrothrix) were investigated at four stations in Sweden and Finland, including deep vertical profiles of anoxic sediment layers, to assess their presence and activity under different environmental contexts.

**Results:**

Using metatranscriptomic data for rRNA-based community profiling and gene expression combined with porewater geochemistry, evidence of abundant and active cable bacteria was found, peaking below 20 cm depth in deep anoxic sediment layers of Koljö Fjord on the Swedish West Coast. This zone coincided with elevated gene expressions related to sulphide oxidation (including *sqr*) and nitrate reduction (*napA*), as well as an abundant presence of sulphide and a sharp nitrate peak. Phylogenetic analyses revealed a diverse assemblage of *Ca*. Electrothrix includes several potential novel taxa. The co-occurrence of cable bacteria activity, sulphide availability, and a nitrate peak at depth suggests that these organisms may be supported by local nitrate production under anoxic conditions.

**Conclusions:**

Our findings challenge the prevailing view that cable bacteria are restricted to shallow sediment horizons and demonstrate their activity and diversity in deep, anoxic layers. This expands the known ecological niche of cable bacteria and suggests that locally produced nitrate under anoxic conditions may facilitate their activity at depth. This discovery advances our understanding of ecology in anoxic marine environments, providing new insights into marine cable bacteria, sediment biogeochemistry, and analogues of early Earth microbial ecosystems.

**Supplementary Information:**

The online version contains supplementary material available at 10.1186/s40793-026-00895-7.

## Background

Cable bacteria are multicellular filamentous organisms characterised by a distinct metabolism that spatially couples the oxidation of sulphide (H_2_S) with the reduction of oxygen (O_2_) or nitrate (NO_3_^−^) by channelling electrons through their filaments over cm-scale distances [1–3]. These bacteria belong to the *Desulfobulbaceae* family and are traditionally classified into two genera: *Ca.* Electrothrix and *Ca.* Electronema for marine and freshwater sediments, respectively [4]. However, recent findings indicate a substantially greater diversity of potentially up to ninety species divided among six genera [5].

The consensus is that cable bacteria are restricted to the upper few cm of surface sediments, where O_2_ or NO_3_^−^ are abundant, while H_2_S is available in close proximity [3, 6]. Cable bacteria possess a metabolic strategy based on long-distance electron transport, giving them a competitive advantage over other sulphur (S)-oxidising bacteria [7]. Their presence in sediments is influenced by a range of environmental parameters, such as bottom water redox conditions, bioturbation [8, 9], salinity and temperature [10], which collectively control the activity, abundance, and diversity of these organisms. Ecologically, their importance is rooted in regulating redox gradients, trace metals [11], nutrient cycling [12], generating a firewall against euxinia [13], and influencing the behaviour of other microbial communities [14, 15].

Cable bacteria have been found in a diverse range of aquatic environments, including rivers, estuaries, coasts, and salt marshes, across freshwater, brackish, and marine settings [8, 16, 17]. Thus far, their presence deep in anoxic sediment layers has not been documented, highlighting the importance of particular environmental conditions for their growth and activity.

This study reveals that metabolically active cable bacteria, hereafter referred to as marine cable bacteria (*Candidatus* Electrothrix), thrive deep in anoxic sediment layers potentially sustained by NO_3_^−^ pockets, challenging the conventional view that they are restricted to surface sediments. These findings offer new insights into their ecological and biogeochemical significance, particularly in removing H_2_S from deeper layers.

## Materials and methods

### Study area and sediment collection

**Fig. 1:**
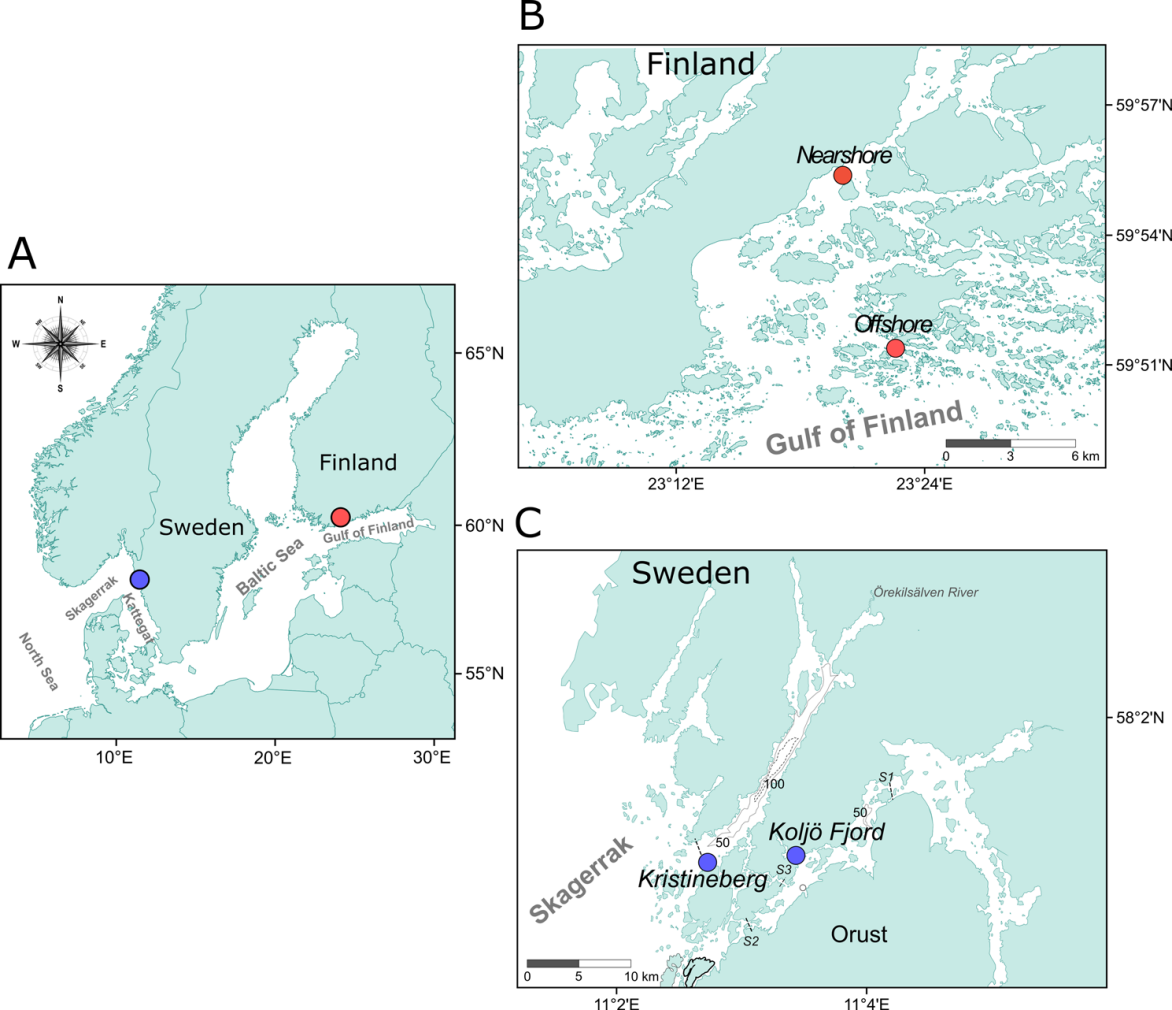
Maps of the study areas. (**A**) Map of the Baltic Sea. The two dots designate the study areas. (**B**) Tvärminne Archipelago in southern Finland, with stations marked as Nearshore and Offshore. **(C)** Swedish West Coast, with stations marked as Kristineberg Bay and Koljö Fjord, and the adjacent sills: Havsten Fjord (S1), Skagerrak (S2), and Gullmar Fjord (S3)

Sediments from Kristineberg Bay and Koljö Fjord on the Swedish West Coast (Fig. 1A, C) were collected aboard *R/V Alice*. Data from two contrasting stations in the Tvärminne Archipelago in southern Finland (Fig. 1B) from another study were used due to the availability of a comparable dataset [18].

**Fig. 2:**
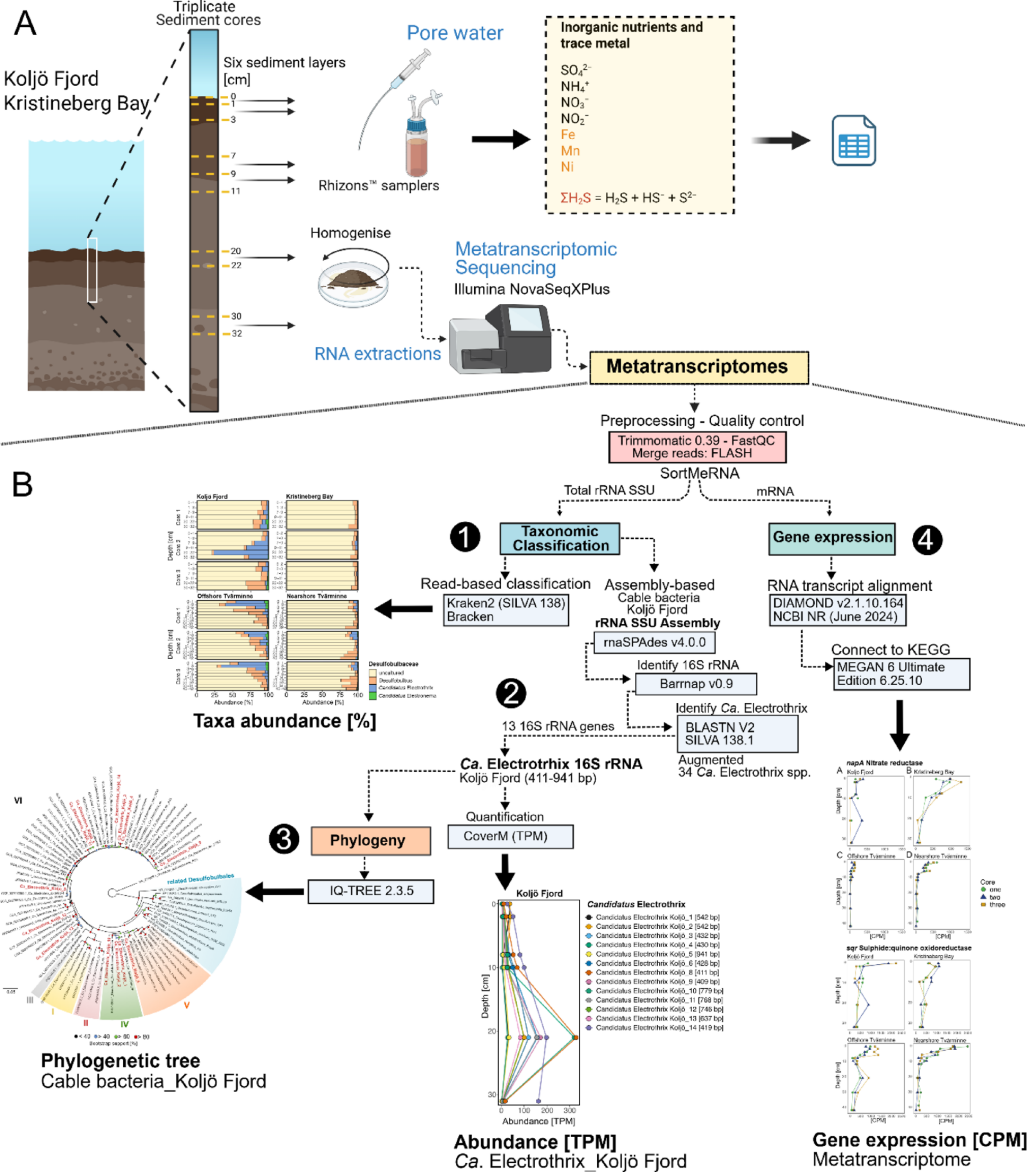
Overview of the sampling and analysis workflow. (**A**) Sediment collection from Koljö Fjord and Kristineberg Bay and the main analyses performed. (**B**) Data analysis workflow following RNA sequencing of metatranscriptomes, leading to taxonomic classification using read-based (1) and assembly-based (2) approaches, as well as phylogenetic analysis of cable bacteria (3) and gene expression analysis (4)

Triplicate sediment cores (Core #1, Core #2, and Core #3) were collected at each site using the Gemini Twin Corer, which retrieves a maximum of two cores per deployment. Therefore, two separate casts were required: Cores #1 and #2 from the first cast and Core #3 from the second. Sediment cores were sliced at depths of 0–1, 1–3, 7–9, 9–11, 20–22, and 30–32 cm for RNA sequencing (metatranscriptomic) and porewater analyses (Fig. 2A). For Kristineberg Bay cores #2 and #3, the uppermost intervals were modified to 0–2 and 2–3 cm instead of 0–1 and 1–3 cm, with all other depths samples as previously described. The separated sediment layers were then placed into 215 mL polypropylene containers (Noax Laboratory, Product No. 207.0215PP). After each layer was homogenised, a 2–3 mL sample of the mixed sediment was transferred to a 15 mL centrifuge tube. These samples were promptly flash-frozen in liquid nitrogen and stored at −80 °C until RNA extraction was performed.

The studied stations cover a range of bottom water redox conditions, from oxic sites (Kristineberg Bay and Tvärminne Nearshore) to hypoxic or anoxic ones (Koljö Fjord and Tvärminne Offshore) (Fig. S1 and Table S1). These classifications are based on standard O_2_ thresholds: ≥ 2 mg L^−1^ (≥ 63 µM) for oxic, ≤ 2 mg L^−1^ (≤ 63 µM) for hypoxic, and 0 mg L^−1^ (0 µM) for anoxic conditions [19]. Notably, Koljö Fjord experiences variable deep-water renewal, resulting in hypoxic or anoxic bottom waters at the time of sampling [20].

### Porewater collection and treatment

Bottom water and porewater samples were extracted using Rhizons™ (0.12–0.18 μm pore size) and collected into 10 mL polyethylene syringes as described in Hermans et al. [18]. Two parallel porewater series were extracted from each core. The first series was designated for total alkalinity, sulphate (SO_4_^2−^), inorganic nutrients (NH_4_^+^, NO_3_^−^, and NO_2_^−^), and trace metals, including iron (Fe), manganese (Mn), and nickel (Ni), whereas the second series was reserved for total sulphide analysis (ΣH_2_S = H_2_S + HS^−^ + S^2−^). Subsamples for trace metal analysis were acidified with distilled nitric acid. Syringes for ΣH_2_S analysis were prefilled with 1 mL zinc acetate (10%) to trap ΣH_2_S as ZnS. Subsamples for SO_4_^2−^, ΣH_2_S, and trace metal analyses were stored at 4 °C, whereas subsamples for inorganic nutrients were frozen at − 20 °C.

### Porewater analyses

Dissolved NH_4_^+^ concentrations were determined after the indophenol method using Segmented Flox Analysis (Alpkem SFA, O. I. Analytical Flow Solution IV). Porewater concentrations of NO_3_^−^ and NO_2_^−^ were determined using a carrier stream that facilitates continuous mixing with a buffer solution. First, NO_3_^−^ was reduced to NO_2_^−^ using cadmium. Following the addition of phosphoric acid, NO_2_^−^, both initially present and formed through the reduction of NO_3_^−^, was used to diazotise sulphanilamide in an acidic solution [21]. In a separate batch, NO_2_^−^ concentrations were determined using the same method as described above but without the cadmium reduction step. The concentration of NO_3_^−^ was derived by subtracting NO_2_^−^ from the total (NO_3_^−^ + NO_2_^−^).

Acidified samples were analysed using Inductively Coupled Plasma-Mass Spectrometry (ICP-MS; Thermo Scientific, XSERIES 2 and Agilent 7800) to determine Fe, Mn, and Ni contents. Here, dissolved Fe and Mn are considered as Fe^2+^ and Mn^2+^; however, some Mn^3+^ or colloidal and nanoparticulate Fe and Mn might also be present [20].

Concentrations of sodium (Na), potassium (K), magnesium (Mg), and chloride (Cl) in Koljö Fjord were determined using ion chromatography (IC).

### RNA extraction and sequencing

Total RNA was extracted from 2 g of each homogenised sediment sample using the RNeasy PowerSoil Total Kit (Qiagen). A TURBO DNA-free kit (Invitrogen) was used to eliminate residual DNA contamination in the eluate. At SciLifeLab in Stockholm, libraries were prepared for 36 samples (Supplementary Data 1), 18 for Kristineberg Bay and Koljö Fjord each (three independent biological replicates per sediment depth) using TruSeq Stranded mRNA polyA (Illumina). The purified libraries were subsequently sequenced at SciLifeLab utilising a NovaSeqXPlus platform (NovaSeqXSeries Control Software 1.2.0.28691) with 2 × 150 bp configuration. The sequencing libraries are available in the NCBI repository under BioProject accession number PRJNA1253322.

### Bioinformatics and statistics

#### Read pre-processing and quality control

RNA sequencing libraries (metatranascriptomes) from Kristineberg Bay and Koljö Fjord yielded an average of 101 million reads (Supplementary Data 1). Illumina adapters were removed via targeted primer sequencing [22] through the SeqPrep 1.2 programme with default parameters. The absence of PhiX control sequences was verified by aligning the reads to the PhiX genome (NCBI Reference Sequence: NC_001422.1) using Bowtie 2 V 2.3.5.1 [23]. Quality control was performed using the programmes FastQC V 0.11.9 [24] and MultiQC V 1.12 [25] before and after the trimming step. Trimming was carried out using Trimmomatic 0.39 [26] (LEADING:20, TRAILING:20, MINLEN:80), and Cutadapt V 4.5 [27]. The trimmed reads were merged using FLASH V 1.2.11 [28]. The average number of merged reads was 54% (ranging from 42 to 73%), resulting in a read length of 220 bp per sample. Following the SAMSA2 pipeline recommendations [29], the paired reads were merged with nonpaired forward reads. The methods used for the Tvärminne libraries are explained in detail by Hermans et al. [18].

#### Taxonomic classification by SSU rRNA, differential abundance and correlations

To determine the taxonomic composition of active bacteria at the genus level we followed the method after Broman et al. [30] and Hermans et al. [18]. Firstly, SSU rRNA reads were extracted from the quality-trimmed metatranscriptomic reads using SortMeRNA 4.3.6 with the SILVA database (silva-bac-16s-id90.fasta) [31]. These reads were classified using Kraken2 [32] against the SILVA 138 SSU (June 2024) database and abundance estimates were refined with Bracken to improve quantitative accuracy (Figs. 1 and 2B).

A complementary assembly-based analysis to verify the presence of cable bacteria in Koljö Fjord was conducted (Fig. 2B). Contigs representing partial 16S rRNA gene sequences (13 contigs, 411–941 bp) were assembled, annotated, and quantified from metatranscriptomes (see Supplementary Material; Supplementary Data 2).

Differences in taxon abundance across sediment depths were assessed using Kruskal–Wallis tests followed by Duncan’s post hoc test, with p-values adjusted using the Benjamini–Hochberg false discovery rate correction. Pearson correlations between bacterial abundances, sediment depth, and geochemical variables (e.g., NO₃⁻ and H_2_S) were calculated and visualised using the *corrplot* function from the R package [33]. To further explore the associations between microbial taxa and sediment depth, correlation heatmaps were generated between genus-level taxonomic abundances and depth indicator variables representing each sampled sediment layer (0–1, 1–3, 7–9, 9–11, 20–22, and 30–32 cm) using the *cor_heat* function of the microViz R package [34]. Prior to analysis, taxonomic counts were normalised using the “compositional” transformation implemented in microViz, which converts counts to proportions within each sample to account for differences in sequencing depth. The *cor_heat* function computes Pearson correlation coefficients, which are displayed in the heatmap cells.

The p-values for the correlations were calculated using the *rcorr* function from the Hmisc R package [35] based on the same transformed abundance matrix. The resulting p-values were adjusted for multiple comparisons using the Benjamini–Hochberg false discovery rate correction. Taxon annotation summaries (relative abundance) were visualised alongside the heatmaps using microViz functions.

Co-abundance correlations among taxa were inferred using SparCC (Sparse Correlations for Compositional data) [36] implemented with 100 iterations and significance assessed using two-sided pseudo-p values generated from 100 bootstrap replicates. Correlations were calculated using Dirichlet-normalized bacterial abundance data.

#### Phylogeny of cable bacteria from Koljö Fjord

**Fig. 3:**
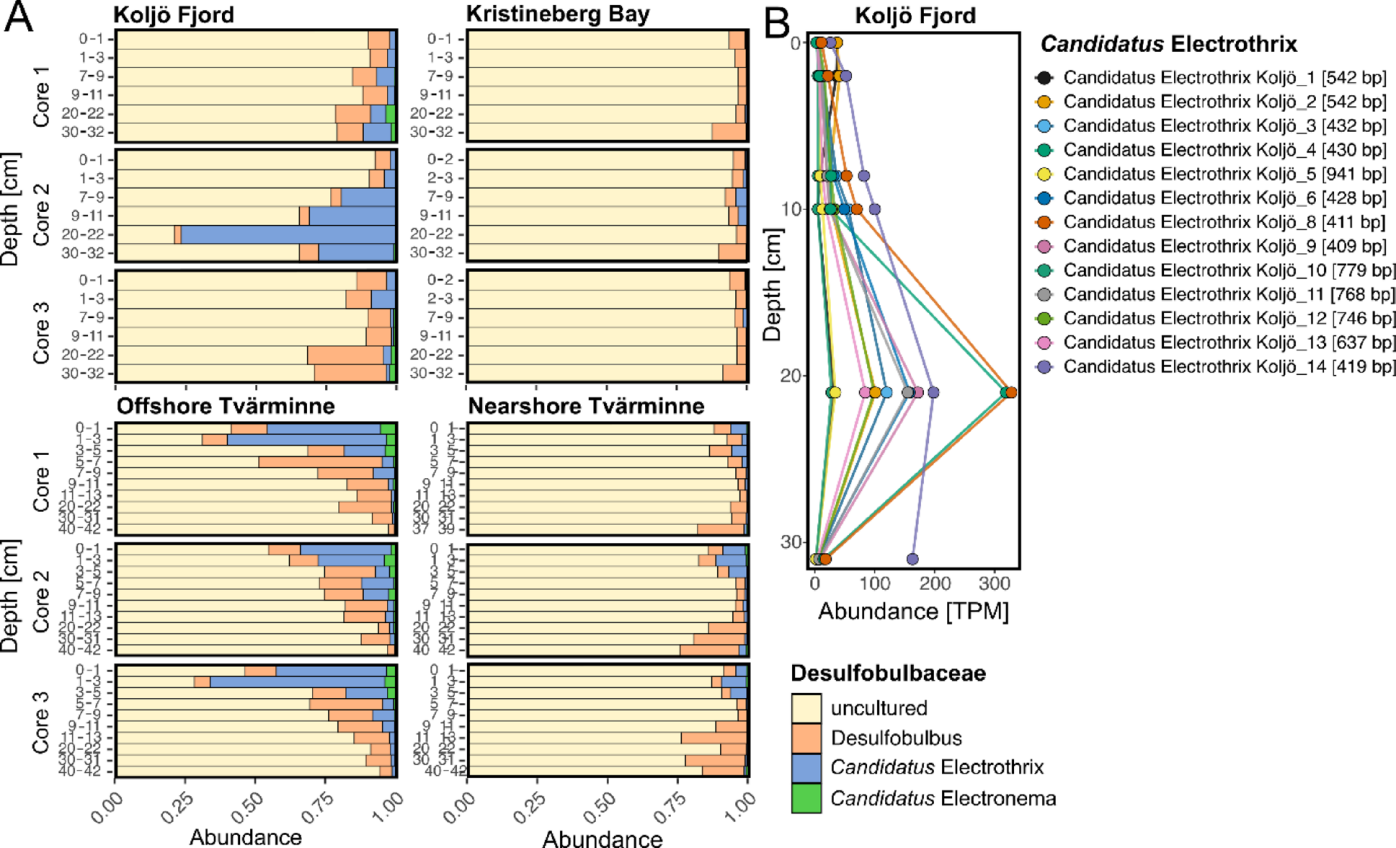
Abundance of *Ca*. Electrothrix in the *Desulfobulbaceae* family. (**A**) Relative abundance of 16S rRNA genes from total SSU rRNA reads, identified using Kraken2 and Bracken (read-base classification) across vertical profiles from 0 to 32 cm at Koljö Fjord and Kristineberg Bay, and up to 42 cm at the Tvärminne Archipelago stations. (**B**) Average abundance of transcript per million (TPM) of 13 partial 16S rRNA gene sequences assigned to *Ca*. Electrothrix by depth in Koljö Fjord. These partial 16S rRNA gene sequences (411–941 bp) were identified after the assembling and annotation of SSU rRNA from Koljö Fjord samples (assembly-based classification). The sequence lengths, in base pairs (bp), are shown in parentheses

To assess the phylogenetic placement of *Ca*. Electrothrix from Koljö Fjord, a 16S rRNA gene phylogenetic tree was constructed (Figs. 2B and 3). Thirteen partial 16S rRNA sequences (411–941 bp) assembled from the metatranscriptomes and annotated as *Ca*. Electrothrix were aligned with 61 reference cable bacteria sequences, 13 related Desulfobulbales, and *Geobacter sulfurreducens* as the outgroup (Supplementary Data 3). Multiple sequence alignment was performed using MUSCLE v5.1.0 [37], and phylogenetic inference was conducted using IQ-TREE 2.3.5 [38] with ModelFinder Plus (MFP+MERGE) for optimal model selection. Branch support was evaluated with 5000 ultrafast bootstrap replicates, SH-aLRT (5000 replicates), and Bayesian-like transformation of aLRT (aBayes). The final tree was visualised using FigTree v1.4.4.

#### Gene expression

**Fig. 4:**
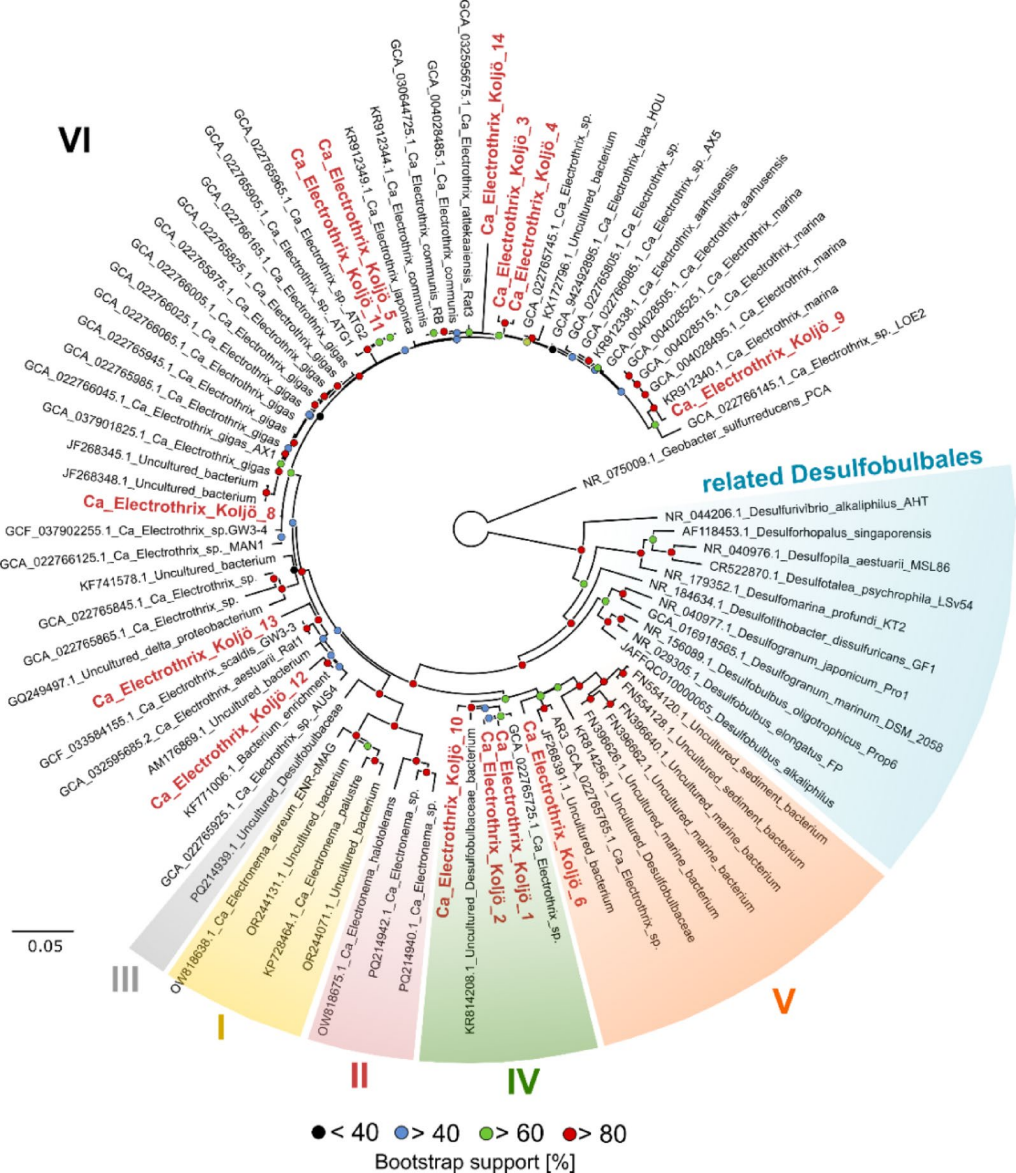
Phylogenetic tree of cable bacteria from Koljö Fjord. Thirteen partial 16S rRNA gene sequences annotated as *Ca*. Electrothrix, from Koljö Fjord, were aligned with 61 reference cable bacteria, 11 related Desulfobulbales, and *Geobacter sulfurreducens* as the outgroup (list of sequences in Supplementary Data 3). Sequence alignment was performed using MUSCLE and phylogenetic inference using IQ-TREE 2.3.5 with ModelFinder Plus (MFP+MERGE). Branch support was evaluated with 5000 ultrafast bootstrap replicates, SH-aLRT (5000 replicates), and Bayesian-like transformation of aLRT (aBayes). Roman numerals indicate cable bacteria clusters as defined by Ley et al. [5]

SortMeRNA 4.3.4 and its provided database (silva-bac-16s-id90.fasta) [31] were used to separate rRNA from total RNA reads. Following Broman et al. [30] and Hermans et al. [18], DIAMOND v2.1.10.164 was utilised to categorise the non-rRNA reads against the NCBI NR database (retrieved on June 3, 2024) by applying an 1e−^10^. The daa-meganizer tool included in MEGAN 6 Ultimate Edition 6.25.10, built 27 June 2024, was used to eliminate eukaryotic and viral data and connect the diamond results to the KEGG database: megan-map-Feb2022 [39, 40]. A MEGAN file containing absolute counts was generated using the MEGAN tool for computer comparison. This file was then imported into MEGAN software to extract all KEGG KO classifications. Sequence counts were normalised to counts per million (CPM; calculated as relative proportion × 1 million) (Figs. 2B and 4). A comprehensive matrix of all KEGG classifications can be found in Supplementary Data 4.

## Results

### Abundance of cable bacteria

Read-base taxonomy classification of 16S rRNA (SSU) gene sequences identified 3,119 bacterial genera (370,937,895 sequences) in Koljö Fjord, 3,055 (453,889,324 sequences) in Kristineberg Bay, 3,339 (871,131,658 sequences) at the Offshore station, and 3,124 (564,362,145 sequences) at the Nearshore station in the Tvärminne Archipelago (Supplementary Data 2). Proteobacteria and Desulfobacterota were the dominant phyla across the data, accounting for 36% and 19% of the total abundance, respectively (Fig. S2). Within the *Desulfobulbaceae* family (the most abundant family and genera in Fig. S3 and S4), undescribed forms were dominant across all stations (1.34%), followed by *Desulfubulbus* (0.09%) and cable bacteria; *Ca*. Electrotrix and *Ca*. Electronema (0.08 and 0.01%).

However, the abundance of *Ca*. Electrothrix at the Offshore station and Koljö Fjord (Fig. 3A and Fig. S5) accounted for 0.11% of the total abundance, corresponding to 416,616 and 937,608 sequences, respectively. In contrast, Kristineberg Bay and the Nearshore station (with decreasing abundance toward deeper layers) exhibited negligible abundances of 0.02% (115,717 sequences) and 0.05% (316,115 sequences), respectively.

The vertical distribution of marine cable bacteria at the Offshore station in the Tvärminne Archipelago clearly decreased with depth. The relative abundances (within the *Desulfobulbaceae* family) ranged from 32.3 to 40.4% at 0–1 cm and 23.6–62.3% at 1–3 cm, dropping sharply to 0.7–1.4% at 20–22 cm. In contrast, at Koljö Fjord, the abundance of cable bacteria increased with depth, from average relative abundances of 2.3% at 0–1 cm and 5.0% at 1–3 cm to 28.2% at 20–22 cm and 12.6% at 30–32 cm. The maximum observed abundance of *Ca*. Electrothrix reached 76% in core #2 (edgeR p-value of 0.05 for the difference between the 0–1 cm and 20–22 cm layers). Thus, cable bacteria were highly abundant in core #2 and, to a lesser extent, in core #1 from the same cast. In contrast, core #3 from a different cast exhibited low abundance.

The presence of *Ca*. Electrothrix in Koljö Fjord was further supported by the assembly-based taxonomy analysis (Fig. 2B). Using this approach, thirteen partial 16S rRNA gene sequences (13 contigs, 411–941 bp) assigned to *Ca*. Electrothrix were recovered (Supplementary Data 2). The resulting abundance profiles (Fig. 3B, Fig. S6) were consistent with those from the read-based taxonomy classification using Kraken2 + Bracken (Fig. 3A).

Average abundances were 12 and 18 TPM at 0–1 and 1–3 cm depths, respectively, with a pronounced maximum at 20–22 cm (141 TPM on average; up to 328 TPM for *Ca*. Electrothrix Koljö_8). While the Kruskal‒Wallis test indicated significant differential abundances between depths (p-value < 0.05), the pairwise Dunn test revealed significant differences between 1 and 3 and 30–32 cm depth (p-value < 0.05) (Table S3). The comparison between 1 and 3 cm and 20–22 cm depth yielded a p-value of 0.01 (adjusted p-value = 0.07). The results revealed that various cable bacteria types were present deep in the anoxic sediment layers of Koljö Fjord, with the highest abundance found between 20 and 22 cm.

### Several species of *Ca.* Electrothrix reside deep within anoxic sediment layers

A phylogenetic tree based on the 13 partial 16S rRNA genes revealed several *Ca.* Electrothrix types in Koljö Fjord (Fig. 4). According to the recent cable bacteria phylogeny [5], nine sequences were assigned to Cluster VI, which included species such as *Ca.* Electrothrix communis and aarhusiensis.

These species are known from Baltic Sea sediments and other brackish or marine environments.

The 16S rRNA gene sequences of *Ca.* Electrothrix from Koljö Fjord appeared to represent novel species. Although *Ca.* Electrothrix Koljö_9 aligned closely with *Ca.* Electrothrix marina, as evidenced by two high-quality BLASTN alignments with 99% identity (219 bp and 196 bp, E-values of 6e^− 10^ and 7e^− 94^), suggesting a close evolutionary relationship.

Three sequences, Koljö_1, Koljö_2, and Koljö_10, belong to Cluster IV, which includes cable bacteria from the North Sea and a mud volcano in Costa Rica. These data suggest that cable bacteria from Koljö Fjord consists of multiple evolutionary lineages, some likely novel.

### Co-abundance of cable bacteria with S-oxidisers in Koljö Fjord

**Fig. 5:**
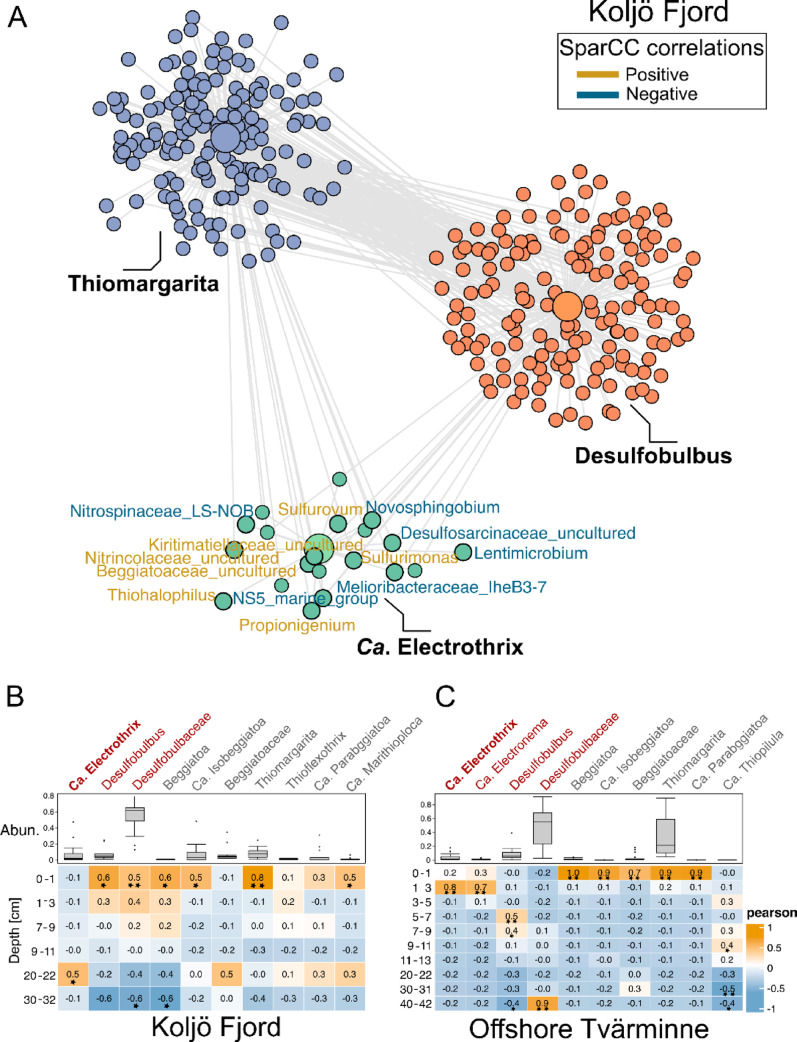
Co-abundance and correlations of cable bacteria. (**A**) SparCC co-abundance network from Koljö Fjord, organised into three colour-coded clusters (green, orange, and blue) representing distinct bacterial communities that significantly correlate with each focal taxon: *Ca*. Electrothrix, *Desulfobulbus*, and *Thiomargarita*, which are highlighted as focal taxa (three largest central nodes). All non-focal nodes in each cluster contain bacteria from diverse lineages that are significantly correlated (*p* < 0.05) with at least one focal taxon, calculated on Dirichlet-normalized bacterial abundance data. The edges (gray connecting lines) represent co-occurrence relationships, whereas the text colour of the genus names, in the *Ca*. Electrothrix cluster, indicates the correlation direction: gold = positive and blue = negative. (**B, C**) Pearson correlation coefficients (within each box) for the ten most abundant *Desulfobulbaceae* and *Beggiatoacea* taxa across sediment depths in Koljö Fjord (**B**) and offshore Tvärminne (**C**), were calculated between compositional-transformed genus-level abundances and sediment depth indicator variables. Single and double asterisks indicate *p* < 0.05 and corrected *p* < 0.05 (Benjamini-Hochberg; Supplementary Data 2). Boxplots represent the relative abundance of each taxon

Previous studies have identified large colourless S-oxidising bacteria, such *Beggiatoaceae*, as potential competitors of cable bacteria due to their similar metabolic requirements [8, 9]. In Koljö Fjord, *Beggiatoaceae* accounted for 0.32% of the total microbial community, and *Ca*. Thiomargarita was the most abundant genus (0.07%). SparCC co-abundance network analysis of *Ca*. Electrothrix, *Ca*. Thiomargarita, and *Desulfobulbus* (Fig. 5A) revealed distinct ecological niches, such as *Ca*. Electrothrix shared fewer associations with the other two taxa, which were more strongly interconnected. Notably, four of the seven significant (*p* < 0.05) co-abundance correlations involving *Ca*. Electrothrix were with S-oxidising bacteria, including *Sulfurovum*, *Sulfurimonas*, *Thiohalophilus*, and an unclassified member of *Beggiatoaceae*, with (R² = 0.56–0.63), suggesting potential functional interactions deep in sediments (Fig. 5A).

Most *Beggiatoaceae* genera identified in Koljö Fjord (Fig. 5B) and the Offshore station (Fig. 5C) were associated with surface layers (1–3 cm), as was also observed in Kristineberg Bay (*Beggiatoaceae* abundance of 0.09%) and the Nearshore station (*Beggiatoaceae* abundance of 0.3%) (Fig. S7). In contrast, positive correlations were observed between *Ca*. Electrothrix and sediments depths of 20–22 cm in Koljö Fjord (R² = 0.5, *p* < 0.05; Fig. 5B). Two *Beggiatoaceae* genera (*Ca*. Parabeggiatoa and *Ca*. Marithioploca) also appeared at this depth, but their abundance was negligible (0.03% and 0.007%, respectively). Similarly, undescribed bacteria of the *Beggiatoaceae* were positive correlated with 20–22 cm in Koljö Fjord.

### Gene expression associated with NO_3_^−^ reduction, H_2_S oxidation, and Ni

**Fig. 6:**
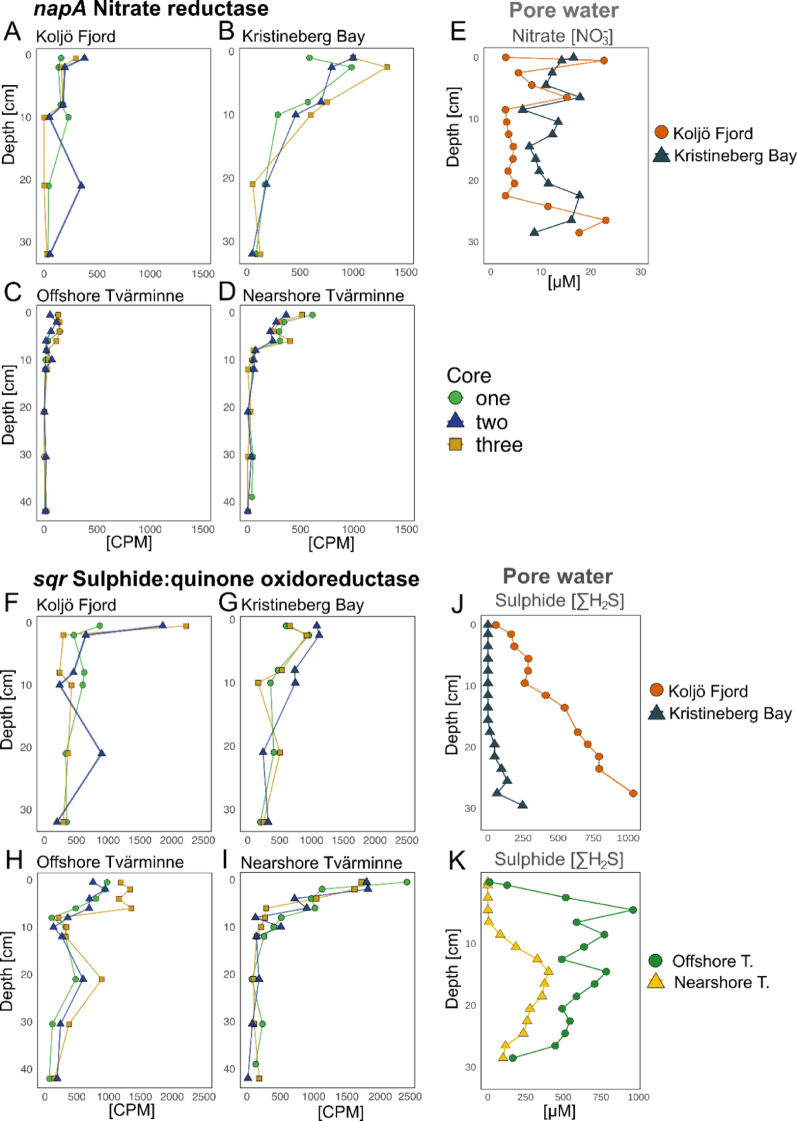
Gene expression and porewater signatures of NO_3_^−^ and ΣH_2_S. Plots **A**–**D** show *napA* gene expression (periplasmic NO_3_^−^ reduction) at community-level, in counts per million (CPM). Plots **F**–**I** display *sqr* gene expression, encoding sulphide quinone oxidoreductase (SQR) involved in H_2_S oxidation. Plot **E** shows NO_3_^−^ concentration profiles, and **J**-**K** show ΣH_2_S porewater profiles

The expression of the marker gene for periplasmic NO_3_^−^ reduction *napA* was highest (~ 500 CPM) at the surface (0–1 cm) and below 20 cm depth in Koljö Fjord (Fig. 6A). Conversely, no strong signal of gene expression was detected at other stations at depths less than 20 cm (Fig. 6B–D). This elevated gene expression at 20–22 cm in Koljö Fjord (gene count matrix in Supplementary Data 4), observed only in core #2, is consistent with the 16S rRNA gene abundance pattern of cable bacteria (Fig. 3A and B). Although the gene expression results here are community-based, an independent analysis showed some specific taxa-expressions of the *napA* gene, such as from *Ca*. Electrothrix aestuarii, which showed higher expression at 20–22 than surface layers (see Supplementary Material and Fig. S8).

High expression of sulphide: quinone oxidoreductase (*sqr*) involved in ΣH_2_S oxidation was found at Koljö Fjord at 20 cm depth (Fig. 6F). The highest expression of the *sqr* gene was detected at 0–1 cm (~ 2000 CPM) and 20–22 cm depth (~ 1000 CPM) in core #2. Similarly, the Offshore station showed an increasing *sqr* gene expression at 20 cm depth (Fig. 6H). In contrast, Kristineberg Bay and the Nearshore station showed a decreasing *sqr* gene expression toward deeper sediment layers (Fig. 6G, I).

Expression of genes potentially involved in Ni metabolism, such as the ABC.PS.S transporter, increased with depth and coincided with the Ni peak below 20 cm in Koljö Fjord (Fig. S9).

### Porewater profiles

At Koljö Fjord, a porewater NO_3_^−^ peak of ~ 23 µM was found between 20 and 30 cm depth, similar to the concentration at the surface (~ 22.5 µM). The zonation of this peak was considerably lower than the typical depth of oxic nitrification (Fig. 6E). Kristineberg Bay had higher background NO_3_^−^ concentrations than Koljö Fjord, with a slight increase in deeper layers. In Koljö Fjord, porewater ΣH_2_S concentrations (Fig. 6J) increased with depth, reaching a maximum of 1036.8 µM at 27–28 cm, with an average concentration of 475.2 µM. Similar concentrations were found at the Offshore station, with a maximum of 963.5 µM (4–5 cm) and an average of 487.7 µM, albeit having different patterns (Fig. 6K). In contrast, Kristineberg Bay presented a lower concentration (average of 68.1 µM and a maximum of 499.5 µM), similar to the Nearshore station (average of 171.3 and a maximum of 402.9 µM).

**Fig. 7:**
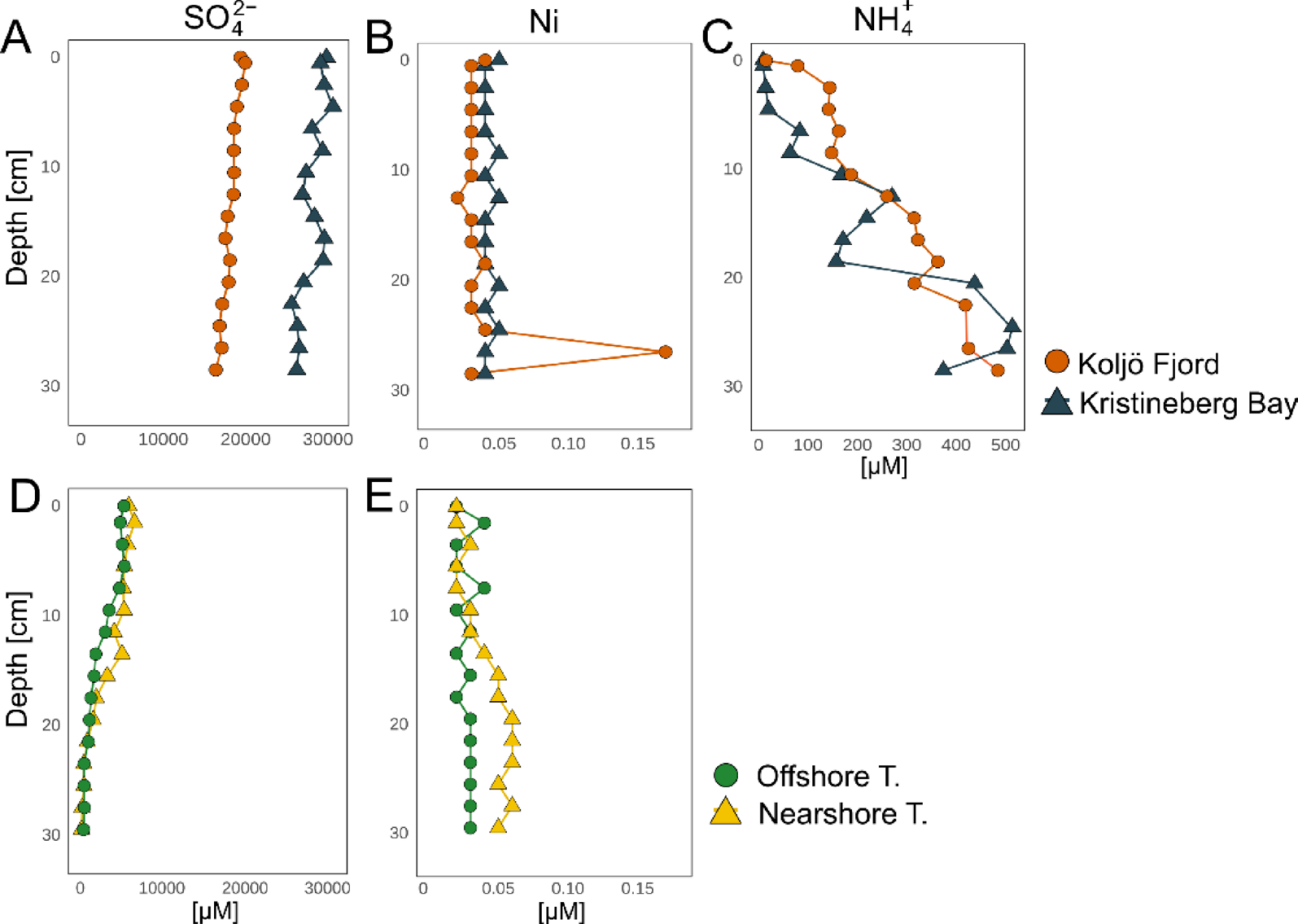
Porewater depth profiles of SO_4_^2−^, Ni and NH_4_^+^. Plots **A** and **D** show SO_4_^2−^ concentration profiles. Plots **B** and **E** show Ni concentration profiles, and **C** shows NH_4_^+^ concentration profiles

Porewater concentrations of SO_4_^2−^ were higher in Koljö Fjord (average of ~ 18.2 mM and maximum of ~ 19.9 mM) and in Kristineberg Bay (average of ~ 27.5 mM and maximum of ~ 30.6 mM) (Fig. 7A) than in the Tvärminne stations (Fig. 7D), where the concentration decreased in deeper layers (average of ~ 2.3–3.3 mM and maximum of ~ 5.4–6.6 mM).

Strikingly, the depth at which the highest abundance of active cable bacteria in Koljö Fjord was detected (20–22 cm) exhibited an Ni peak that reached 0.17 µM (Fig. 7B). This concentration was higher than the average background level of ~ 0.03 µM at all other depths. Kristineberg Bay (Fig. 7B) and the Tvarminne Archipelago stations (Fig. 7E) have similar concentrations with straight uniform profiles. The NH_4_^+^ concentration profiles of Koljö Fjord and Kristineberg Bay (only available there) were similar, increasing toward deeper sediment layers, with an average of 206.7–206.0 and maximums of 476.2–505.6 µM (Fig. 7C) (Supplementary Data 5).

## Discussion

### Evidence for active cable bacteria deep in anoxic sediment layers

Unexpectedly, a high abundance of active cable bacteria was detected in the anoxic Koljö Fjord [20]. Even more strikingly, the *Ca.* Electrothrix abundance was relatively high between 7 and 9 cm and increased toward deeper sediment layers, reaching its maximum between 20 and 22 cm sediment depth, although it remained quite high between 30 and 32 cm as well.

The discovery of *Ca.* Electrothrix deep in anoxic sediment layers in Koljö Fjord challenges the consensus that cable bacteria are restricted to the oxic–anoxic interface of surface sediments [15]. Cable bacteria have not been extensively studied in anoxic environments. However, recently, they have been found in O_2_-deficient systems [41–43], where cable bacteria might survive when NO_3_^−^ is used as the main electron acceptor. Notably, the *Ca.* Electrothrix abundance in this study increased toward deeper sediment layers, contrary to the typical decrease with sediment depth. The abundances found here align with previously reported abundance ranges found in other natural environments, such as the seasonally anoxic Chesapeake Bay, ranging from 0.04 to 3.6% [9].

A strong advantage of 16S rRNA analysis using metatranscriptomics is that it indicates ribosomal activity, thereby supporting its presence but also ensures that the bacteria are metabolically active [44].

The patchy distribution of cable bacteria, with high abundance in core #2 but not in core #3, points towards microspatial heterogeneity in terms of environmental conditions or colonisation dynamics. Patchy distributions of cable bacteria are well documented and are influenced by a range of local factors, including local O_2_, NO_3_^−^, ΣH_2_S availability, sediment characteristics, and bioturbation [8, 9]. Optical sensor profiling further revealed that colonisation often occurs in favourable microenvironments, underscoring the spatial heterogeneity of cable bacteria populations [45]. Such heterogeneity is a recognised feature of their ecology and does not diminish the representativeness of our findings.

### Phylogeny of cable bacteria and interactions with S-oxidising competitors

The phylogenetic diversity of cable bacteria from Koljö Fjord spans several phylotypes of *Ca*. Electrothrix into different clusters; IV, V, and VI [5], while most of them fell within Cluster VI. This cluster encompasses previously described *Ca.* Electrothrix, including gigas [46], communis, aarhusiensis, marina, japonica [4], laxa [47], rattekaaiensis [48], and antwerpensis [49], among others. *Ca*. Electrothrix communis, aarhusiensis and marina are derived from sulphidic sediments located near the Baltic Sea shore [4]. Notably, *Ca*. Electrothrix communis and aarhusiensis are present in both brackish and marine sediments, including salt marshes. Phylogeny of *Ca*. Electrothrix from Koljö Fjord indicates that it constitutes a community, likely representing novel species with diverse evolutionary phylotypes within cable bacteria. This suggests that cable bacteria have broader diversity, occupying more ecological niches than previously recognised, possibly involving NO_3_^−^ reduction or other cryptic electron transfer processes deep in sulphidic and anoxic sediment layers.

Co-abundance analysis revealed that cable bacteria in Koljö Fjord had a few associations with the *Beggiatoaceae* family, which correlated more with *Desulfubulbus*, indicating a distinct ecological niche. *Beggiatoaceae*, which include large colourless S-oxidising bacteria, are often considered competitors of cable bacteria, yet some studies suggest that mutually beneficial relationships can occur under anoxic conditions [50]. The absence of infauna and bioturbation in Koljö Fjord likely favoured cable bacteria, by e.g., more efficient capacity for NO_3_^−^ or sulphite uptake and utilisation, as heavy bioturbation can supress their growth, and promote the growth of competitors such as *Beggiatoaceae* [8, 9].

*Beggiatoaceae* dominated the surface layers, and some taxa extended deeper, consistent with the ability of genera such as *Beggiatoa* and *Ca*. Marithioploca to store NO_3_^−^ and migrate into sulphidic sediments [51, 52]. The unusual enrichment of *Ca*. Electrothrix in deep anoxic layers, alongside correlations with other S-oxidisers, indicates a potentially more widespread role for H_2_S oxidation in anoxic sediments than previously recognised.

### Locally produced NO_3_^−^ may sustain cable bacteria deep in anoxic sediments

The high NO_3_^−^ peak found below 20 cm in Koljö Fjord is unusual, and similar findings under these conditions are scarce. However, a comparable NO_3_^−^ peak in deep anoxic sediment layers of Loch Duich, an organic-rich marine fjord, has been reported [53]. This NO_3_^−^ peak may supply electron acceptors for supporting cable bacteria respiration in deep sediment layers. The NO_3_^−^ peak in Koljö Fjord is linked to the expression of the *napA* gene, which encodes periplasmic NO_3_^−^ reduction (Fig. 6A). While *napA* expression reflects the whole community, limiting its unambiguous taxonomic assignment, independent analysis detected higher deep expression from *Ca*. Electrothrix aestuarii and other closely related candidates (Fig. S8) in the deep layers. In addition, the depth-specific enrichment of NO_3_^−^reduction transcripts indicates a functional NO_3_^−^ removal pathway that coincides with cable-bacteria activity. This elevated gene expression at 20–22 cm in Koljö Fjord (gene count matrix in Supplementary Data 4) is consistent with the 16S rRNA gene abundance pattern found in core #2 (Fig. 3A). This further supports the notion of a localised, patchy distribution of cable bacteria. Periplasmic NO_3_^−^ reductase *napAB* plays a crucial role in NO_3_^−^ reduction in cable bacteria [54], coupled with ΣH_2_S oxidation [6]. Hence, the availability of NO_3_^−^ in deeper sediment layers may facilitate respiration of cable bacteria in the absence of O_2_.

To understand what drives high NO_3_^−^ concentrations in deeper sediment layers at Koljö Fjord, all potential sources were identified. Input of NO_3_^−^ into deeper sediment layers via groundwater would have been associated with a steep decrease in the salinity gradient with depth due to the inflow of freshwater [55]. However, the stable brackish porewater conditions, as evident by the depth profiles of Na, K, Mg, and Cl, indicate that there is no source of groundwater discharging into the deeper sediment layer (Fig. S10). This implies that the NO_3_^−^ peak found deep into the sediment is locally produced through anaerobic oxidation of NH_4_^+^, which is a microbially mediated process.

Anaerobic oxidation of NH_4_^+^ (Fig. 7C) by Fe oxides (Eq. 1) is unlikely to be a source, as Fe oxides would undergo rapid reductive dissolution upon contact with ΣH_2_S [56], and not reach deeper sediment layers (Fig. S11).

1 $$\:8\mathrm{F}\mathrm{e}{\left(\mathrm{O}\mathrm{H}\right)}_{3}+14{\mathrm{H}}^{+}+\mathrm{N}{\mathrm{H}}_{4}^{+}\:\to\:8{\mathrm{F}\mathrm{e}}^{2+}+21{\mathrm{H}}_{2}\mathrm{O}\:+\mathrm{N}{\mathrm{O}}_{3}^{-}$$

Anaerobic oxidation of NH_4_^+^ by Mn oxides (Eq. 2) is likely insignificant. Although Mn oxides are resistant to reductive dissolution by ΣH_2_S [56], their low abundance in the sediment and low porewater Mn^2+^ concentrations (Fig. S11 and Supplementary Data 5) suggest a minor role.

2 $$\:4\mathrm{M}\mathrm{n}{\mathrm{O}}_{2}\:+\mathrm{N}{\mathrm{H}}_{4}^{+}+6{\mathrm{H}}^{+}\to\:4{\mathrm{M}\mathrm{n}}^{2+}+{\mathrm{N}\mathrm{O}}_{3}^{-}+{5\mathrm{H}}_{2}\mathrm{O}$$

No highly expressed genes associated with Mn or Fe oxidation or with hydrazine dehydrogenase (HDH), which catalyses the final step of NH_4_^+^ oxidation in anammox bacteria using nitrite or nitric oxide [57], were identified.

A potential explanation for NO_3_^−^ production deeper in the sediment is through sulfammox, a microbial process coupling NH_4_^+^ oxidation to SO_4_^2−^ reduction under anoxic conditions (Fig. 7A). Initially found in wastewater treatment [58], it has also been reported in marine sediments, though documented occurrences in natural environments are scarce [59, 60]. In sulfammox, the NH_4_^+^/SO_4_^2−^ molar ratio is regarded as a critical parameter, as it not only influences the rates of NH_4_^+^ oxidation and SO_4_^2−^ reduction but also controls the end product. When the NH_4_^+^/SO_4_^2−^ ratio is ≥ 4, NH_4_^+^ is converted into NO_2_^−^. However, when the NH_4_^+^/SO_4_^2−^ ratio is ≤ 2, NH_4_^+^ is overoxidised to NO_3_^−^ to facilitate the reduction of SO_4_^2−^ [61, 62]. Besides controlling the end product, the NH_4_^+^/SO_4_^2−^ ratio also regulates whether sulfammox or anammox is the preferred metabolic pathway. A reactor experiment demonstrated that sulfammox dominated when the NH_4_^+^/SO_4_^2−^ ratio was less than 1.5. Conversely, anammox became the primary pathway when the NH_4_^+^/SO_4_^2−^ ratio exceeded 1.5 [63]. Considering that at Koljö Fjord, the NH_4_^+^/SO_4_^2−^ ratios in the porewater in the deeper sediment were extremely low (< 0.03), this could point toward sulfammox being the primary pathway with NO_3_^−^ as end product (Eq. 3).

3 $$\:3\mathrm{N}{\mathrm{H}}_{4}^{+}+4\mathrm{S}{\mathrm{O}}_{4}^{2-}+2\mathrm{H}\mathrm{C}{\mathrm{O}}_{3}^{-}\:\to\:3\mathrm{N}{\mathrm{O}}_{3}^{-}+4{\mathrm{S}}^{0}+2{\mathrm{H}}_{2}\mathrm{O}+2\mathrm{C}{\mathrm{O}}_{3}^{2-}$$

Besides the NH_4_^+^/SO_4_^2−^ ratio, the abundant presence of HCO_3_^−^ plays a key role in regulating sulfammox, serving both as a pH buffer and carbon source [64]. However, HCO_3_^−^ concentrations exceeding 16.4 mM can inhibit sulfammox [65]. Notably, the estimated HCO_3_^−^ concentrations from our alkalinity profiles ranged between ~ 2.0 and ~ 7.4 mM (Fig. S12; Supplementary Data 5). This falls within a favourable range for sulfammox given the inhibitory threshold of 16.4 mM Furthermore, the pH conditions in Koljö Fjord are suitable for sulfammox [66], given its known preference for slightly alkaline environments [67].

Although sulfammox was recently discovered and the exact metabolic pathways are unknown [62], various bacteria have been reported to be associated with this process, including *Bacillus benzoevorans* [67], *Bacillus cereus* [68], and *Candidatus* Anammoxoglobus [62]. In our dataset, *Bacillus* was relatively abundant in Koljö Fjord (0.9% of the total rRNA reads peaking at 1.5–2.5% at 20–22 cm; Supplementary Data 2). Similarly, *Sulfurimonas*, another genus suggested to be linked to sulfammox [67], reached up to 2.1% abundance at the same depth and co-occurred with *Ca*. Electrothrix (Fig. 5A). Thus, while the geochemical profiles (favourable NH_4_^+^/SO_4_^2−^ ratio and accumulation of NO_3_^−^ between 20 and 30 cm depth) are consistent with the potential for sulfammox, the role of, e.g., *Bacillus* and *Sulfurimonas* in this process remains speculative. Further studies integrating functional gene or genome-resolved analyses are required to verify whether these organisms contribute to sulfammox in marine sediments.

### S-oxidation in deeper sediment layers and Ni utilisation

The high expression of sulphide: quinone oxidoreductase (*srq*), at community level, observed at Koljö Fjord (Fig. 6F) matches the highest cable bacteria abundance and the NO_3_^−^ peak in Koljö Fjord. SQR facilitates the oxidation of H_2_S in the periplasm to produce polysulphides. Although the mechanisms of energy conservation in cable bacteria are not fully understood, *sqr* has been identified in several cable bacteria genomes and may play a role in S-oxidation [49, 54]. Other genes associated with S-oxidation, such as *aprAB*, *soeA*, and *soxY*, as well as polysulphide reductase (*psrA*), S-reduction genes such as *sat* and *dsrB*, and electron transfer genes such as *cytB* and *cydA*, exhibited high expression at 20–22 cm depth in core #2 in Koljö Fjord (Fig. S9, S13 and S14), suggesting constant H_2_S oxidation. Although no evident SO_4_^2−^ accumulation and ΣH_2_S depletion are observed at the depths where cable bacteria are most abundant, this could be attributed to the possibility that cable bacteria may oxidise relatively small amounts of ΣH_2_S, insufficient to visibly alter the porewater profiles of SO_4_^2−^ and ΣH_2_S. This is consistent with recent studies showing that cable bacteria can thrive at extremely low porewater ΣH_2_S concentrations [43, 69]. In Koljö Fjord, a Ni peak below 20 cm coincided with increased expression of Ni-related genes, including the ABC.PS.S transporter (Fig. S9). While this gene is not exclusive to cable bacteria, Ni is essential for their conductive wires [49], and they can tolerate elevated Ni porewater concentrations (~ 1 µM) [11]. The co-occurrence of high Ni and elevated gene expression thus suggests a potential role of Ni in supporting cable-bacteria activity in Koljö Fjord.

### Proposed novel niche for cable bacteria deep in anoxic sediment layers

**Fig. 8:**
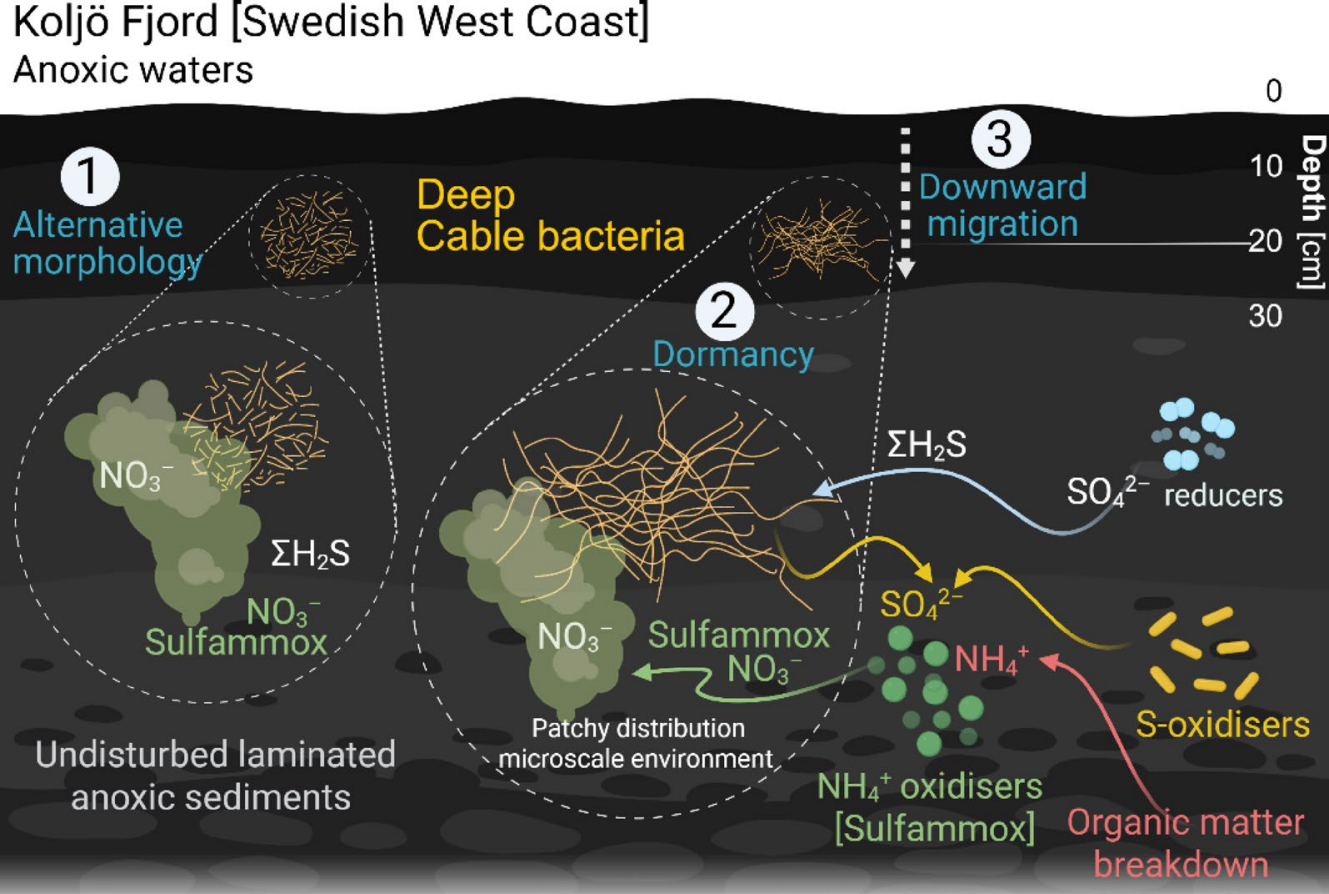
Conceptual model of cable bacteria in deep anoxic sediments. Diagram illustrating three possible scenarios for cable bacteria persistence and activity in deep anoxic sediments, where anaerobic nitrate production (e.g., via sulfammox) generates local NO3 − pockets: (1) local NO3 − availability supporting alternative morphologies, including short or unicellular forms, (2) dormancy and reactivation during episodic NO_2_^−^enrichment, and (3) downward migration toward transient NO3—rich layers

Our model for cable bacteria communities in deep anoxic sediments (Fig. 8) considers sulfammox [59, 60] as a plausible source of NO_3_^−^ (Fig. 6E). We propose that such localised, transient processes may generate microenvironments that enable cable bacteria to access electron acceptors, thereby supporting patchily distributed communities.

The traditional conceptual framework of cable bacteria bioenergetics is based on the spatial arrangement of cells across vertical redox zones, with division of labour among the cells. This traditional model links cathodic O_2_ reduction at the sediment surface to anodic ΣH_2_S oxidation in deeper anoxic layers via electrical currents [54]. However, this study revealed that ΣH_2_S and NO_3_^−^ coexist with cable bacteria within the same deep sediment layers in Koljö Fjord. This scenario suggests a different physiological mode, potentially involving short filaments or even unicellular morphotypes (Fig. 8(1)), thereby reducing the need for conductive nanowires or metabolic differentiation along the filaments. Shorter cable bacterial filaments and fragmented populations have previously been observed in heterogeneous sediment environments and under disturbed redox conditions. For example, short filaments composed of only a few cells have been reported in bioturbated sediments and patchy redox environments [70], and cable bacteria are capable of dynamic filament behaviour, including looping and fragmentation, during motility [71]. Similarly, short filament segments have been observed to transiently explore oxic zones without sustained O_2_ consumption [72]. Although these observations do not demonstrate a direct response to overlapping electron donors and acceptors, they suggest that cable bacterial populations can occur as shorter filament units under heterogeneous redox conditions.

Continuous NO_3_^−^ supply through, for example, sulfammox could support such communities and may represent ancient forms of cable bacteria adapted to O_2_-independent niches. The second possibility is that cable bacteria may remain dormant for extended periods (potentially decades to centuries) until episodic NO_3_^−^ enrichment reactivates them (Fig. 8(2)). Such enrichment could arise not only from local microbial nitrate production but also from episodic physical processes, such as porewater advection or deep-water renewal events that transiently transport nitrate or other electron acceptors into deeper sediment layers. Under such conditions, the sudden availability of electron acceptors could stimulate rapid growth of previously dormant populations. This is consistent with laboratory observations, where sediments stored under anoxic conditions for several months, with a low abundance of cable bacteria (14 m cm^− 2^), developed a thriving community (724 m cm^− 2^) when electron acceptors were provided [69]. The lack of bioturbation in Koljö Fjord as there is no macrofauna present due to persistent anoxia [73], may further facilitate the long-term preservation of intact cable bacteria. An interesting example of long-term preservation in Koljö Fjord is the survival of dinoflagellate cysts, which germinate after remaining in anoxic sediments for up to a century [74].

Alternatively, cable bacteria might gradually migrate downward toward transient NO_3_^−^ rich layers (Fig. 8(3)), as suggested by their peak abundance just above the NO_3_^−^ maximum at 20–22 cm depth. In this case, communities would not reach a steady state but rather respond dynamically to ephemeral NO_3_^−^ pockets.

Future studies should characterise environments similar to Koljö Fjord, to determine whether the deep, anoxic niches of cable bacteria represent a generalised but overlooked phenomenon. Sediment incubations can be used to test for anoxic NO_3_^−^ production, for example, via sulfammox, and explore potential syntrophic interactions between S-oxidisers and nitrifiers. This could help shed light on the mechanisms that sustain cable bacteria activity in deep, anoxic sediments.

## Conclusions

Our findings expand the current understanding of cable bacteria ecology by demonstrating the presence and activity of *Ca*. Electrothrix in deeper anoxic sediment layers of Koljö Fjord. These observations challenge the prevailing view that cable bacteria are restricted primarily to surface sediments and suggest that they may also occur in deeper sediment layers under certain geochemical conditions. This raises the possibility that cable bacteria could contribute to sulphur cycling, including ΣH_2_S turnover, in deeper sediments.

The occurrence of cable bacteria in these anoxic environments further suggests the existence of a deep and spatially heterogeneous community that may occupy ecological niches not previously recognised. These results further prompt questions about syntrophic relationships between cable bacteria and organisms capable of anoxic nitrification, potentially reflecting ecological analogues of microbial communities that may existed in early Earth marine ecosystems.

## Supplementary Information

Below is the link to the electronic supplementary material.

Supplementary Material 1

## Data Availability

All data are available in the main text and in the Supplementary Material. Including supplementary figures and tables in the Supplementary Material file. All raw sequence data are deposited and available online at the NCBI repository under accession number PRJNA1253322 (https://www.ncbi.nlm.nih.gov/bioproject/?term=PRJNA1253322). The main scripts and procedures for data analysis performed in the study are publicly available in the GitHub repository: https://github.com/Alexis-Fonseca/CB_Koljo_Fjord.git. Supplementary Excel documents labeled as Supplementary Data are available on figshare.

## Abbreviations

*Ca.*: *Candidatus*
Ca: Calcium
Cl: Chloride
Fe: Iron
H_2_S: Sulfide
HS^−^: Hydrosulfide
IC: Ion chromatography
ICP-MS: Inductively coupled plasma-mass spectrometry
K: Potassium
Mn: Manganese
Na: Sodium
*napA*: Periplasmic nitrate reductase
NH_4_^+^: Ammonium
Ni: Nickel
NO_2_^−^: Nitrite
NO_3_^−^: Nitrate
O_2_: Oxygen
S-oxidizers: Sulfide-oxidizers
S^2−^: Sulfide ion
SO_4_^2−^: Sulfate
*sqr*: Sulfide: quinone reductase
ΣH_2_S: Total sulfide
