# Supplementary material for: Evidence for cable bacteria inhabiting deep in anoxic sediment reveals a novel ecological niche
Source: Environ Microbiome. 2026 Apr 15;21:54. doi: 10.1186/s40793-026-00895-7 (PMC13081432; doi:10.1186/s40793-026-00895-7)
Supplement: Supplementary file 1 — Supplementary Material 1 [file 40793_2026_895_MOESM1_ESM.docx]

Supplementary Materials for

**Evidence for cable bacteria inhabiting deep in anoxic sediment reveals a novel ecological niche**

Alexis Fonseca^1,2^*, Martijn Hermans^1^*, Francisco J.A. Nascimento^1,2^, Christian Stranne^1,3,4^, Alf Norkko^5^, Bo G. Gustafsson^1,6^ & Christoph Humborg^1^

^1^Baltic Sea Centre, Stockholm University, Stockholm, Sweden

^2^Department of Ecology, Environment and Plant Sciences, Stockholm University, Stockholm, Sweden

^3^Department of Geological Sciences, Stockholm University, Stockholm, Sweden

^4^Bolin Centre for Climate Research, Stockholm University, Stockholm, Sweden

^5^Tvärminne Zoological Station, University of Helsinki, Hanko, Finland

^6^Baltic Nest Institute, Stockholm University, Stockholm, Sweden

*Corresponding authors. Email: [alexis.fonseca@su.se](mailto:alexis.fonseca@su.se) and [martijn.hermans@su.se](mailto:martijn.hermans@su.se)

## Hydrography and redox classification of sampling sites Commonly used thresholds to classify bottom water redox conditions are (Algeo and Li, 2020): oxic >2 mg L⁻1 (>63 μM), hypoxic <2 mg L⁻1 (<63 μM), and anoxic 0 mg L⁻1 (0 μM).

Kristineberg Bay is fully marine and has oxic bottom waters. Koljö Fjord is part of an open-ended fjord system encompassing the Orust and Tjörn Islands (Fig. 1C). It is restricted by three shallow sills connected to the adjacent Havsten Fjord: (S1) at a water depth of 12 m, Skagerrak (S2) at a water depth of 8 m, and Gullmar Fjord (S3) at a water depth of 5 m (Fig. 1D). Hydrography is controlled by the inflow of brackish surface water through Kattegat–Skagerrak, originating from the Baltic Sea. Freshwater input is of minor importance as no major riverine inputs are discharged into the fjord. The deep waters of Koljö Fjord undergo renewal. However, reoxygenation may not be guaranteed because of its variable frequency, which ranges from annual to several years (Paul et al., 2023). At the time of sampling, the bottom waters of Koljö Fjord were hypoxic/anoxic.

The two stations in the Tvärminne Archipelago exhibited relatively low salinities, although they remained brackish. However, they share similar hydrographical characteristics and sediment structures as the two stations situated on the Swedish West Coast. These stations are designated as Nearshore and Offshore (Fig. 1B). The Nearshore station has oxic bottom waters and is located close to the outflow of the Pojo Bay estuary, whereas the Offshore station has hypoxic bottom waters, as it is situated in a small enclosed bay further offshore the archipelago.

While the Offshore station is not situated completely offshore but rather farther out at sea, the term "offshore" is used to distinguish this station from the nearshore station.


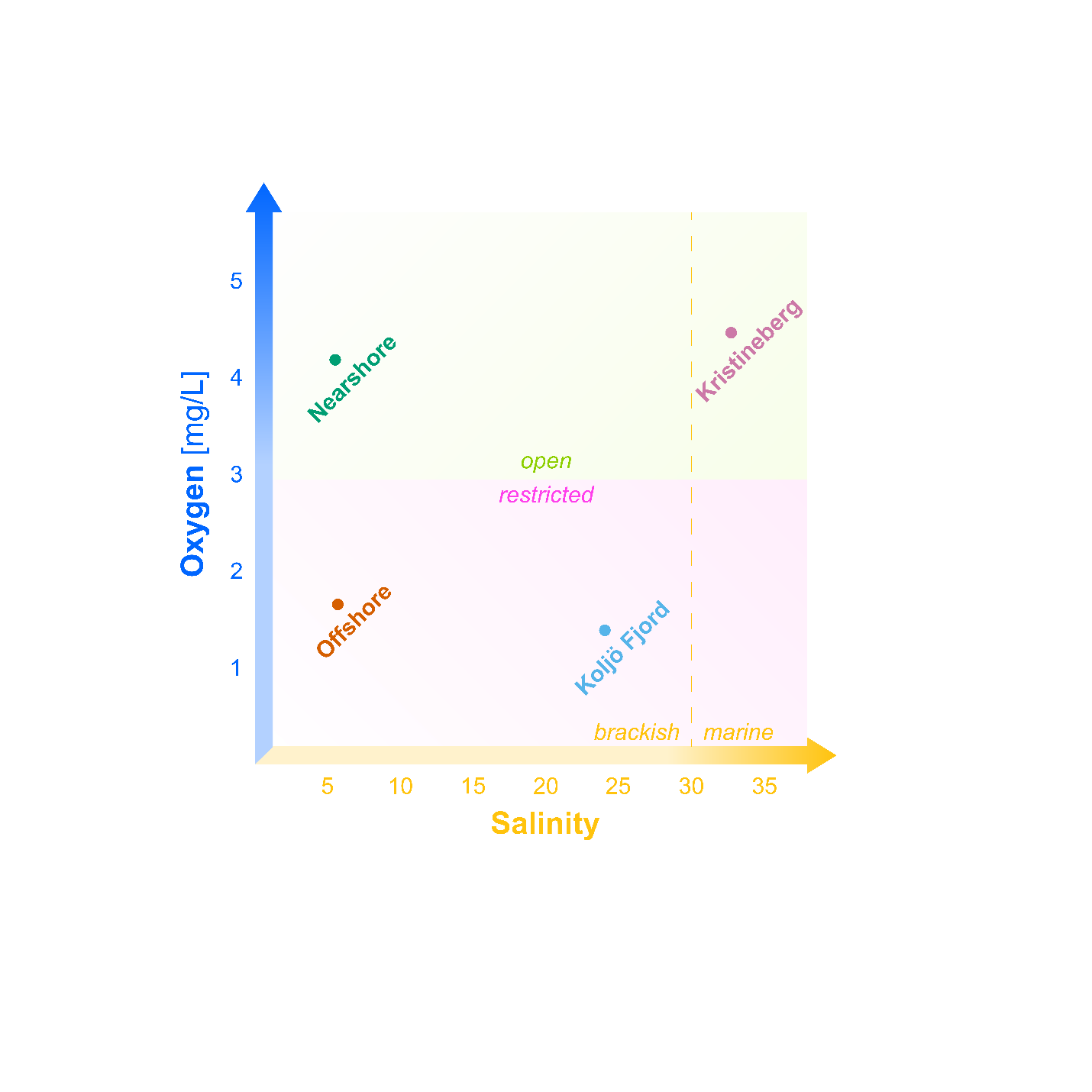


Figure S1 | Classification of the stations by salinity and O_2_ concentration. Classification of stations based on bottom water characteristics: O_2_, salinity at the time of sampling, and physical configuration (open vs. restricted).

## Assembly-based taxonomy classification and quantification of cable bacteria in Koljö Fjord

In contrast to the read-based taxonomic classification, which provides a community-level overview, assembly-based analysis was performed to recover and quantify longer *Ca*. Electrothrix 16S rRNA sequences with higher taxonomic resolution. Thus, to further identify and quantify cable bacteria in Koljö Fjord, rRNA gene reads extracted with SortMeRNA were assembled using rnaSPAdes (SPAdes v4.0.0; Bushmanova et al., 2019) with k-mer sizes of 31, 55, 77, 97, and 127. The resulting 16S rRNA gene sequences were identified using Barrnap v0.9 (https://github.com/tseemann/barrnap) and aligned against the SILVA_138.1_SSURef_NR99 database, which was supplemented with 34 *Ca*. Electrothrix 16S rRNA gene sequences were obtained. Alignments were performed using BLASTN v2.15.0+ (Chen et al., 2015) with the parameter -max_target_seqs 1.

Sequences matching *Ca*. Electrothrix spp. with >90% identity, e-value < 1×10⁻¹⁰, and query coverage >95% were retained for quantification. Read mapping of the original rRNA gene dataset against the assembled and annotated *Ca*. Electrothrix 16S rRNA sequences were analysed using CoverM v0.7.0 (Aroney et al., 2025) in “contig” mode, using the TPM method (-m tpm). This analysis recovered 13 partial Ca. Electrothrix 16S rRNA gene sequences ranged from 411 to 941 bp in length (Supplementary Data 2). These 13 rRNA 16S gene sequences serve later to build the *Ca*. Electrothrix phylogeny analysis in Koljö Fjord.


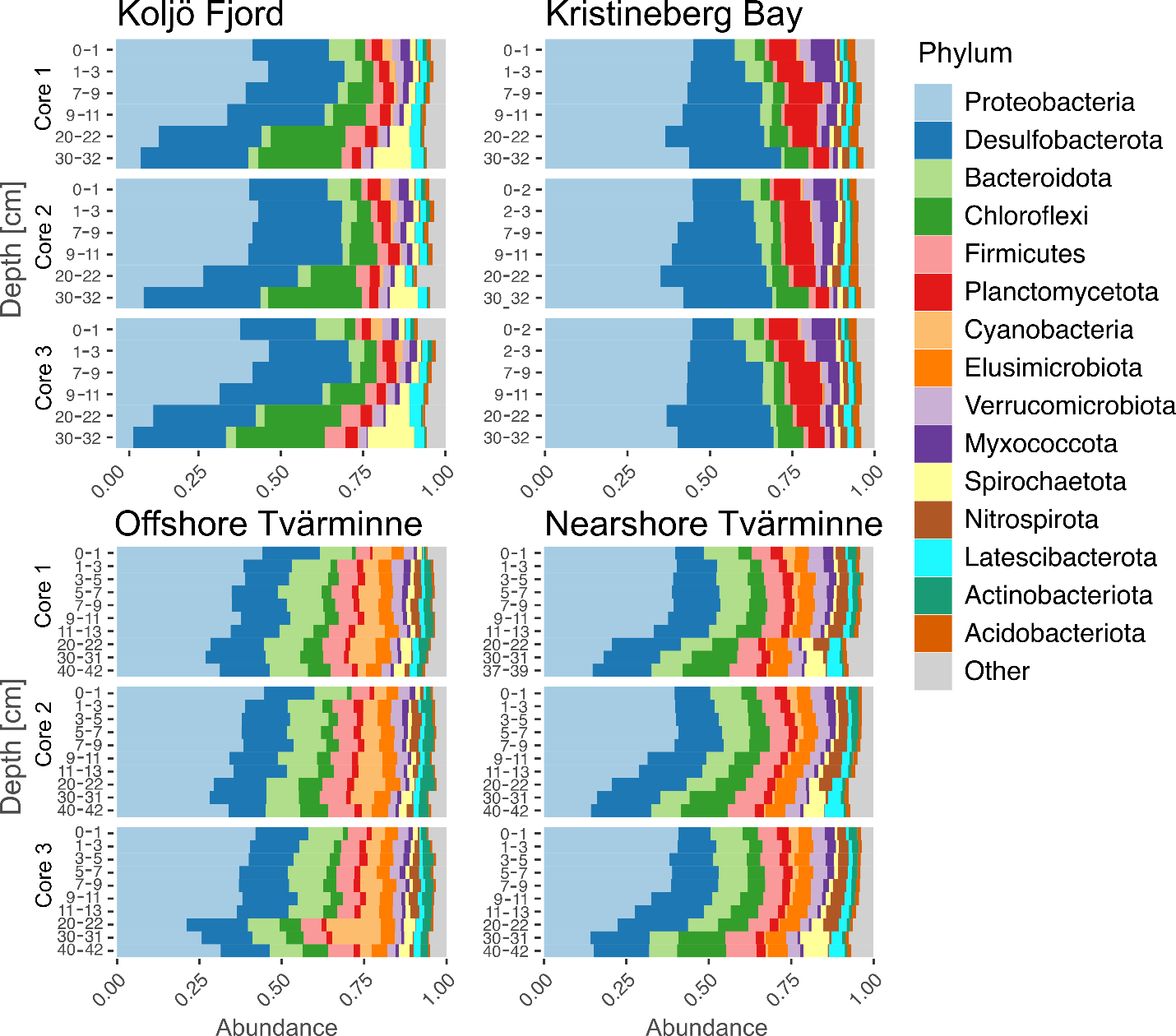


Figure S2 | The 15 most abundant phyla by depth and location. The taxa are ranking from the most abundant to less and deploy in relative abundance.


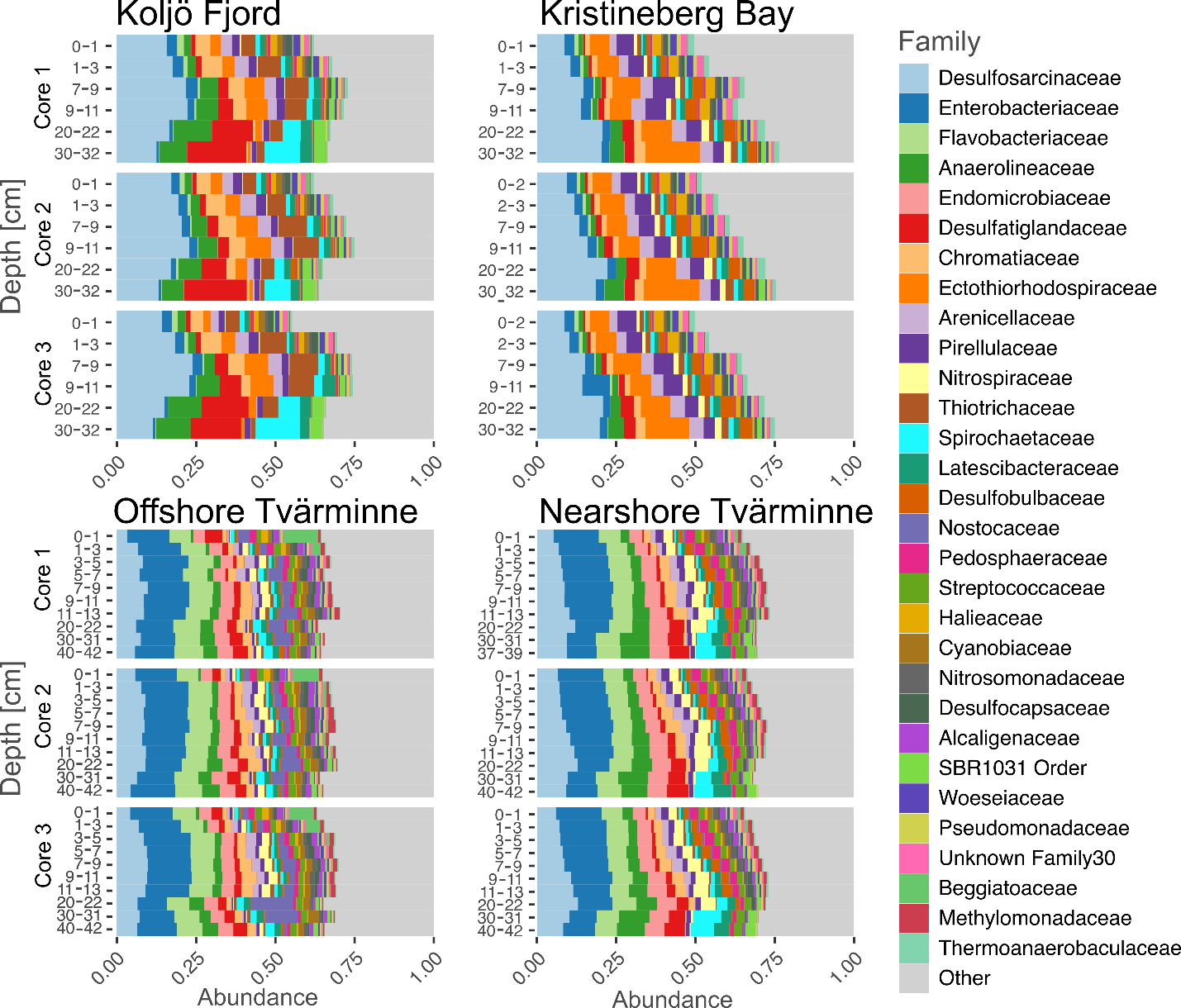


Figure S3 | The 30 most abundant families by depth and location. The taxa are ranking from the most abundant to less and deploy in relative abundance.


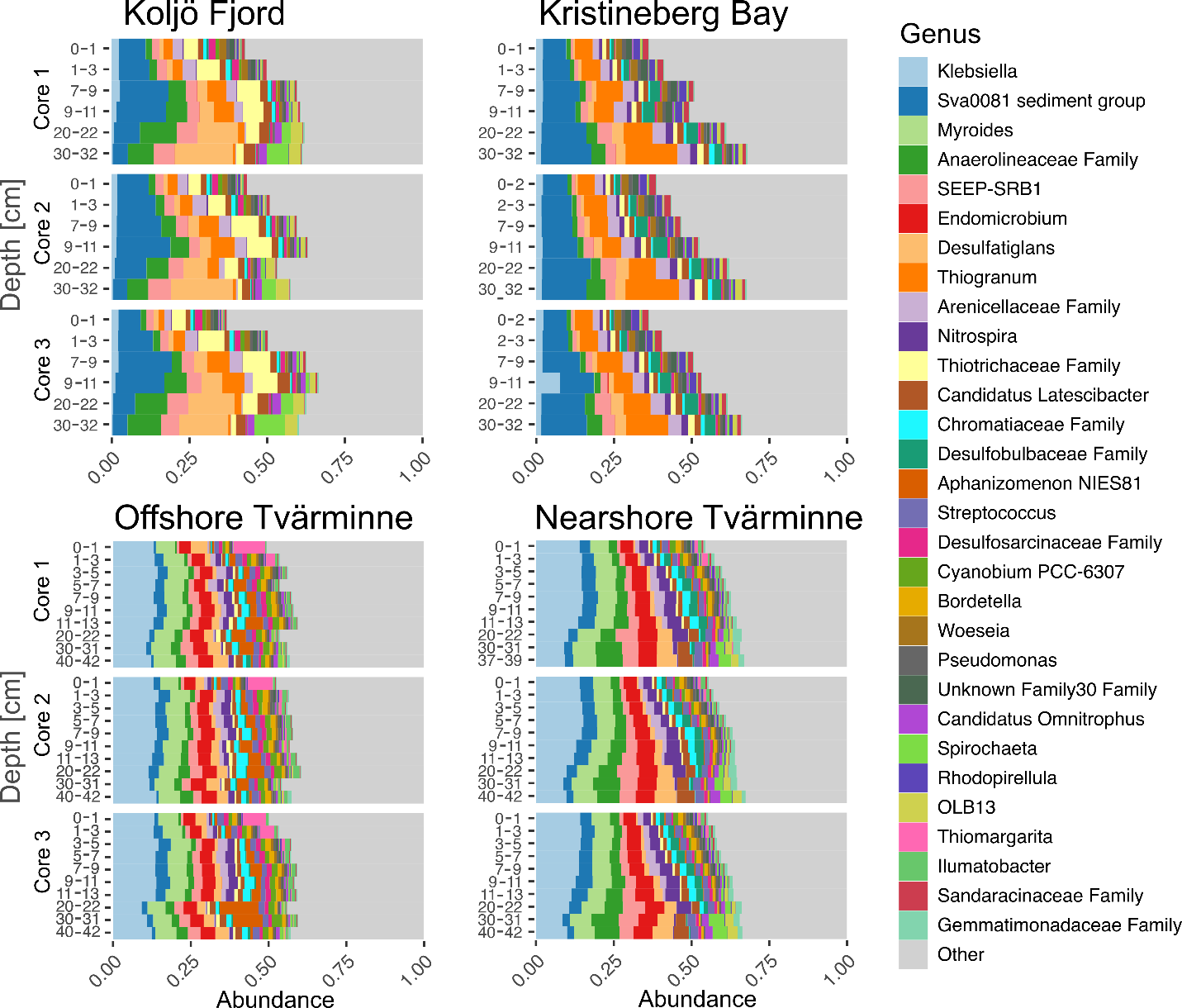


Figure S4 | The 30 most abundant genera by depth and location. The taxa are ranking from the most abundant to less and deploy in relative abundance.


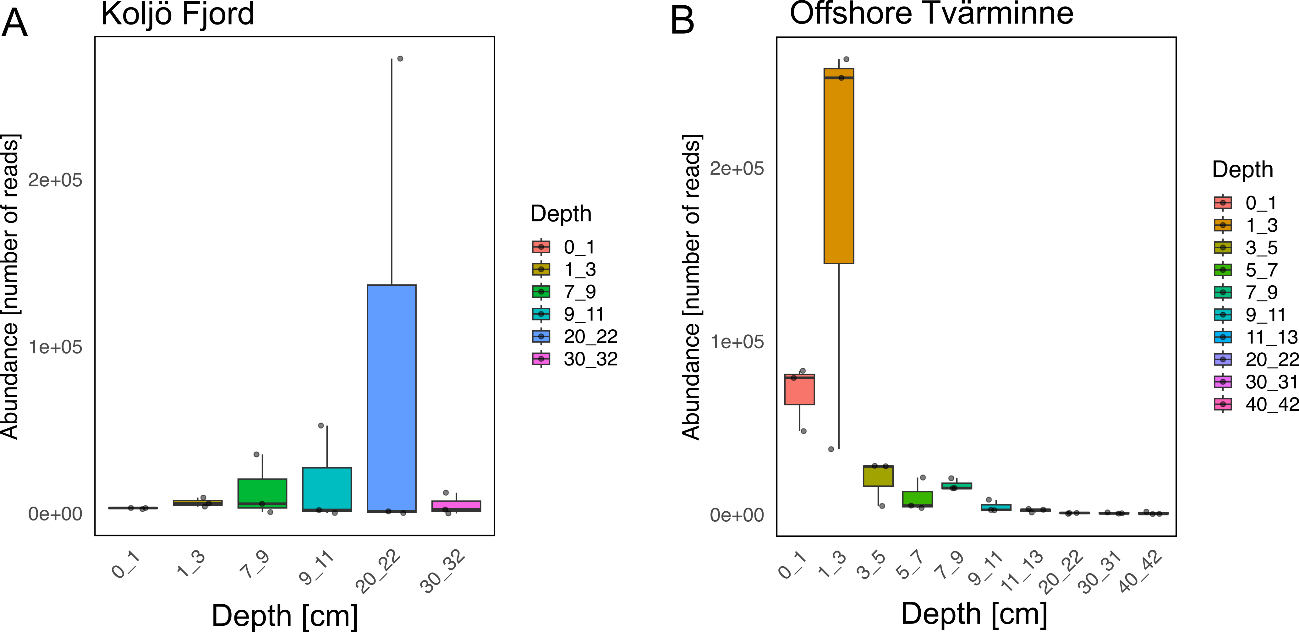


Figure S5 | *Candidatus* Electrothrix abundance as assigned by Kraken2 + Bracken using SSU 16S rRNA genes in Köljo Fjord and Offshore Tvärminne. The 16S rRNA gene sequences were identified through read-based taxonomy classification at (A) Koljö Fjord and (B) the Offshore Tvärminne station.


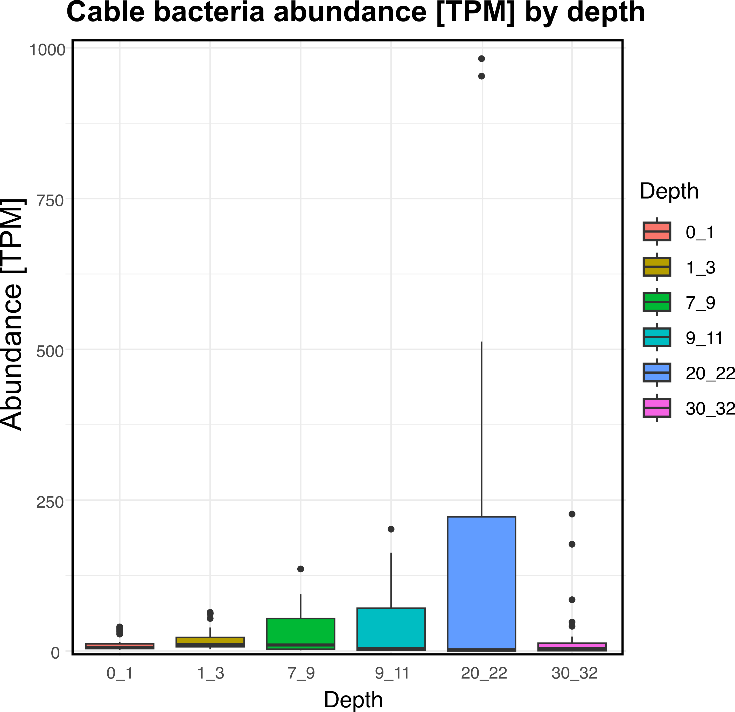


Figure S6 | Abundance in transcript per million (TPM) by depth of partial 16S rRNA genes (13 contigs, 411-941 bp) assigned to *Candidatus* Electrothrix in Köljo Fjord through assembly-based taxonomy approach.


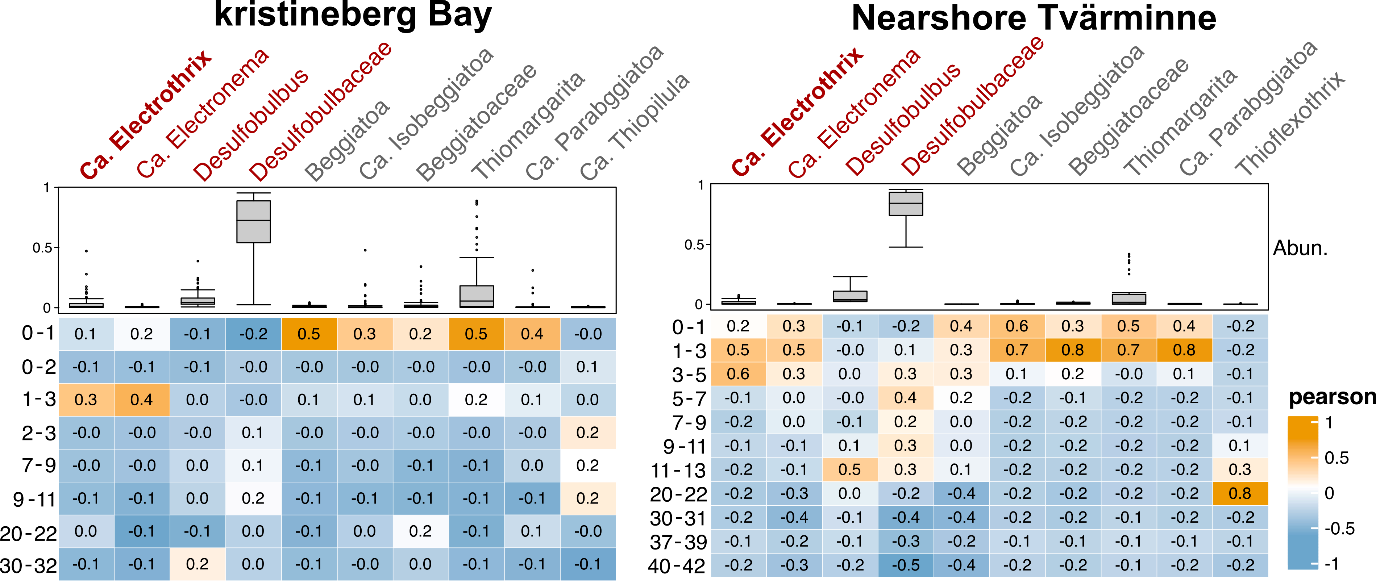


Figure S7 | Pearson correlations. Correlations among depths and the abundance of *Candidatus* Electrothrix and three other genera from the *Desulfobulbaceae* family (indicated in red) and the six most abundant genera from the *Beggiatoaceae* family in Kristineberg Bay and the Nearshore Tvärminne station.

**Independent *napA* gene expression analysis in Koljö Fjord**

To investigate the taxonomic affiliation and expression of periplasmic nitrate reductase (*napA*), we performed independent gene-level analyses. Metatranscriptomic reads from Koljö Fjord were co-assembled using MEGAHIT v1.2.9, and protein-coding sequences were predicted using Prodigal v2.6.3 in metagenomic mode. Translated proteins were screened with HMMER v3.4 against the TIGRFAM model TIGR01706 (*napA*; GA threshold) and re-scanned under relaxed criteria (E < 1e−3) to detect divergent homologs. Candidate NapA proteins were filtered by alignment coverage (>60%) and sequence length (>500 aa) and verified by BLASTP (E < 1e−5) against the NCBI nr database. Candidate sequences were aligned with reference NapA sequences from *Candidatus* Electrothrix genomes (for example, *Ca*. Electrothrix marina, *Ca*. Electrothrix communis, *Ca*. Electrothrix aestuarii, among others), and related nitrate-reducing or sulphur-oxidising taxa (for example, *Beggiatoa*, *Thiomargarita*, *Desulfobulbus*, and *Desulfotalea*) using DIAMOND (blasp) v2.1.11, keeping hits with %identity ≥ 30% and alignment coverage ≥ 50%. Multiple sequence alignment was performed using MAFFT v7, and a maximum-likelihood phylogeny was inferred using IQ-TREE2 v2.4.0 with the ‘-m MFP -B 1000 -alrt 1000 -T 15 -nt AUTO’ commands and 1,000 ultrafast bootstraps. Expression (TPM) was estimated using CoverM v0.7.0 from Bowtie2-mapped reads, and differential expression across depths was tested with edgeR (FDR < 0.05).

The elevated expression of the *napA* gene coincided with a pronounced nitrate peak at 20–22 cm depth in Koljö Fjord (Fig. 6A; gene count matrix in Supplementary Data 4), consistent with the strong transcriptional signal of *Ca*. Electrothrix was observed in core #2 (Fig. 3A–B). However, because metatranscriptomic data reflect community-level expression, the taxonomic origin of *napA* transcripts cannot be unambiguously resolved. To address this, we conducted independent phylogenetic and expression analyses (Fig. S8). The resulting *napA* phylogeny and TPM abundance profiles indicated contributions from multiple taxa, including *Beggiatoa*-related sequences and several candidates identified from the Koljö Fjord metatranscriptome assembly. While some *napA* sequences clustered near the known *Ca*. Electrothrix references, many of which could not be assigned to a single lineage with confidence.


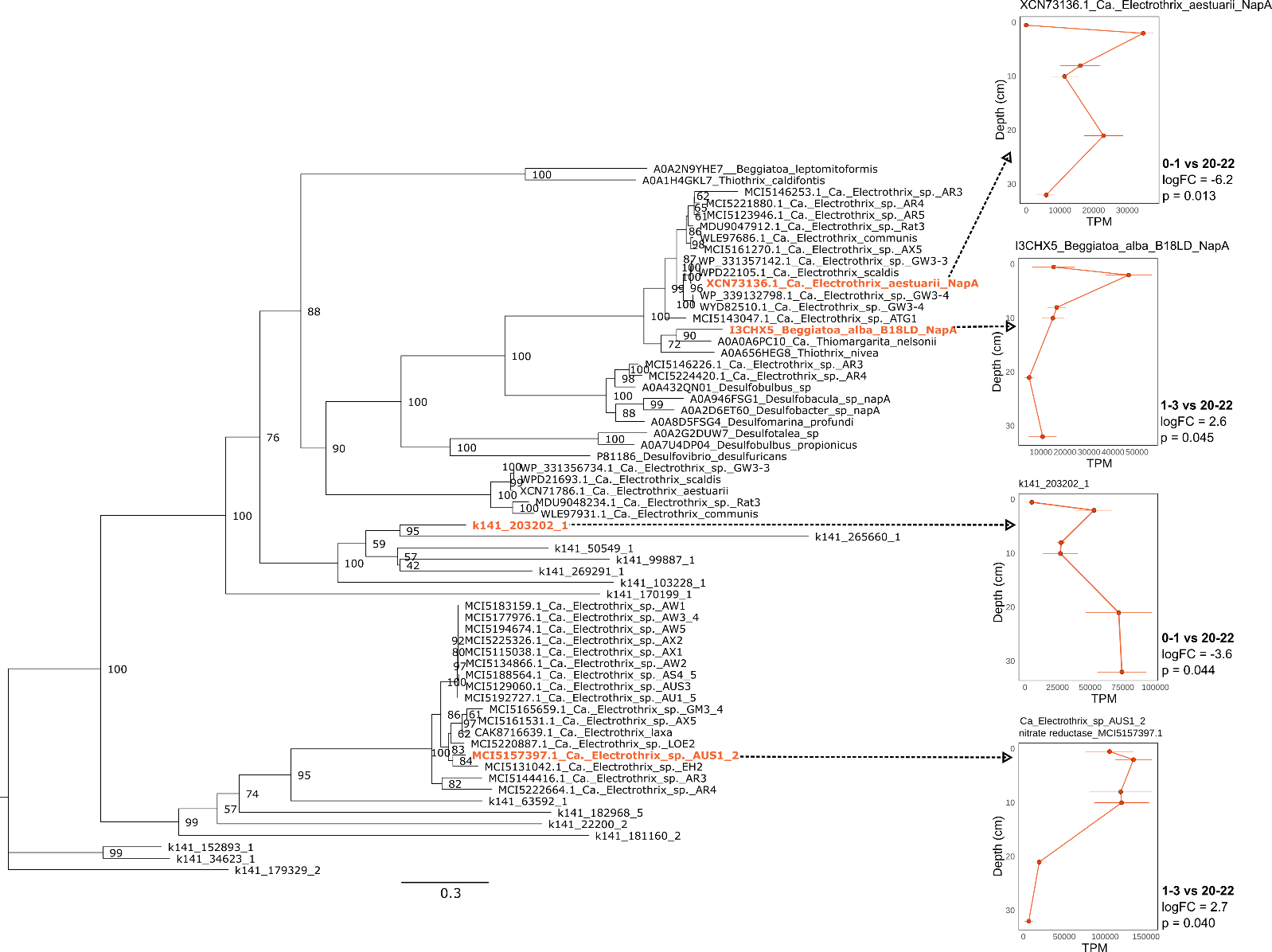


Figure S8 | Phylogenetic analysis and expression profiles of nitrate reductase (*napA*) in Koljö Fjord. Left: Phylogenetic tree of *napA* protein sequences, including 27 *Ca*. Electrothrix references, 21 related nitrate-reducing taxa (*Beggiatoa*, *Thiomargarita*, *Desulfobacter*, etc.), and 14 sequences were identified from the Koljö Fjord metatranscriptome using the *napA* Hidden Markov Model (TIGR01706) as a reference. Sequence annotation was performed using DIAMOND with the same database employed for phylogenetic reconstruction. Right: Transcripts per million (TPM) profiles for all sequences; those showing significant differential expression between surface (0-3 cm) and deep (> 20 cm) sediment layers (edgeR, p < 0.05) are highlighted in red.

Differential expression analysis using edgeR revealed that *Ca*. Electrothrix aestuarii exhibited significantly higher *napA* expression at 20–22 cm compared to the surface layers. Similarly, the assembled *napA* transcript, K141_203202_1, showed a comparable depth-dependent increase (p<0.05). In contrast, *Beggiatoa alba* and *Ca*. Electrothrix sp. AUS_2 displayed significantly higher *napA* expression (p<0.05) in the surface layers (Fig. S8, left panel). The other *napA* sequences showed no significant differences (p>0.05). These results suggest that while depth-resolved *napA* expression can be partially resolved to specific taxa, complete disentanglement of gene-to-taxon relationships remains limited by the complexity of the sedimentary microbial community.


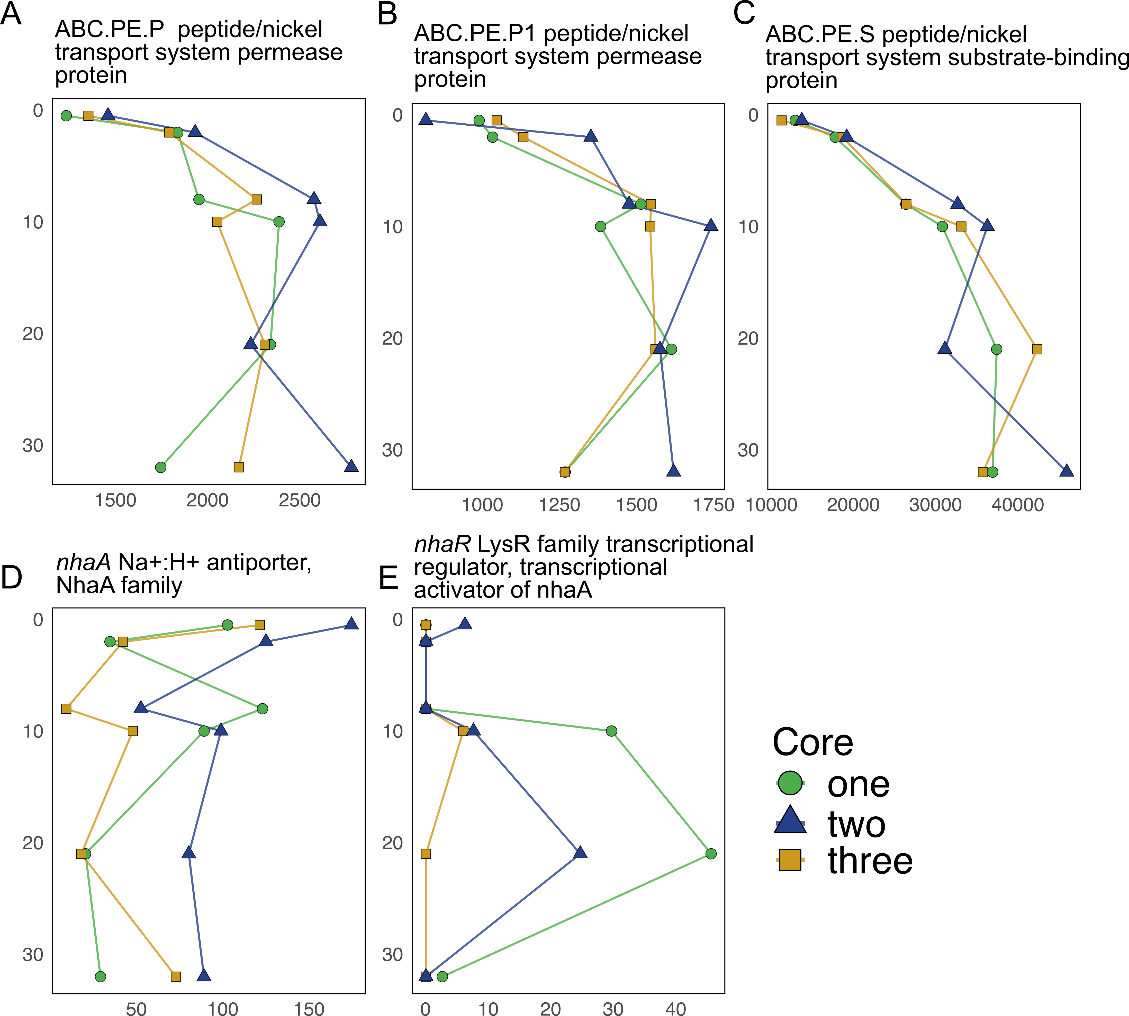


Figure S9 | Gene expression profiles linked to nickel and marine adaptation in Köljo Fjord. A-C) Gene expression profiles by depth of genes associated with nickel metabolism and D-E) marine adaptation.


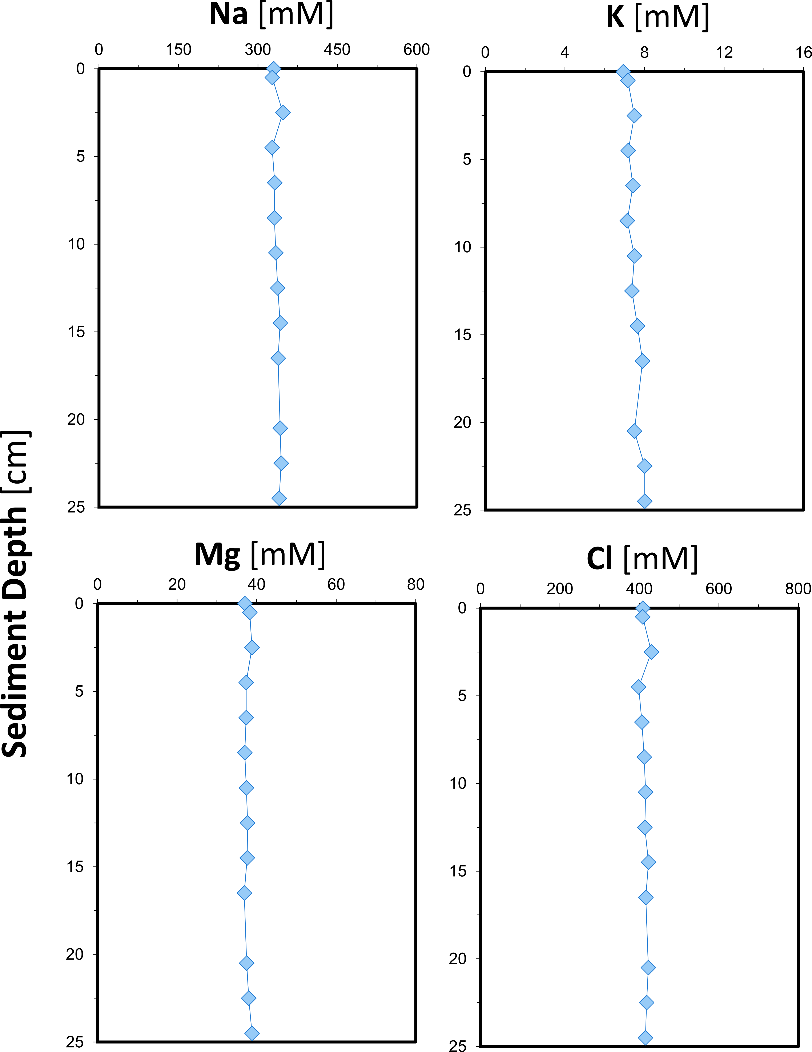


Figure S10 | Porewater profiles of Sodium (Na), potassium (K), magnesium (Mg), and chloride (Cl) concentrations (mM) by sediment depth in Koljö Fjord.


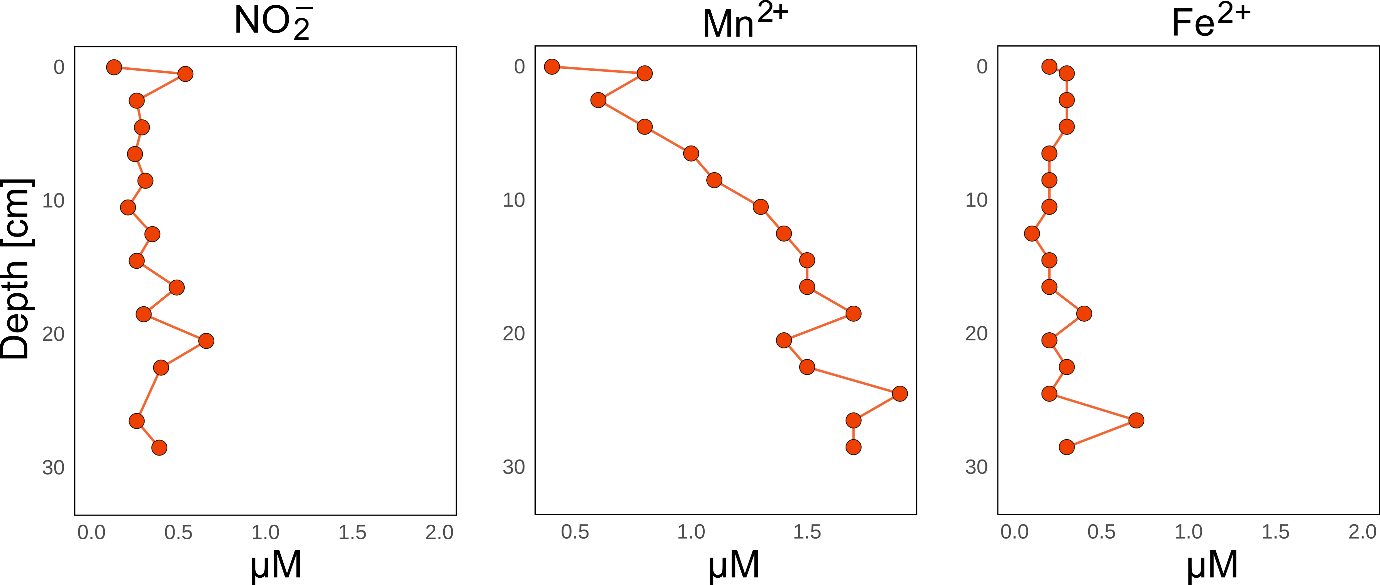


Figure S11 | Porewater profiles of nitrate (NO_2_^−^), manganese (Mn^2+^) and iron (Fe^2+^) concentrations in Koljö Fjord.


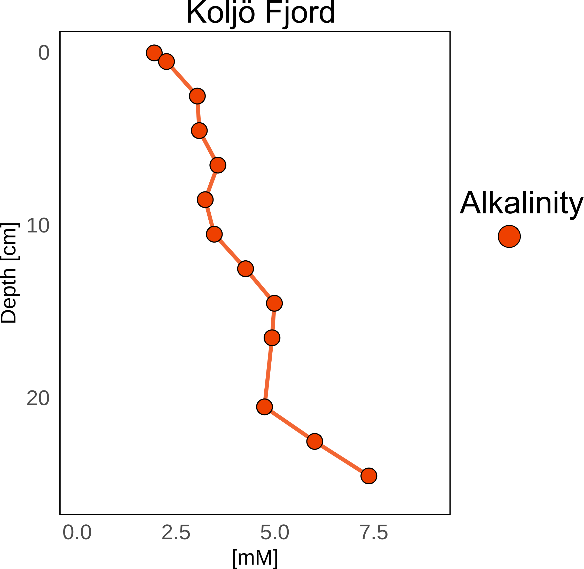


Figure S12 | Porewater profiles of alkalinity concentrations in Koljö Fjord.


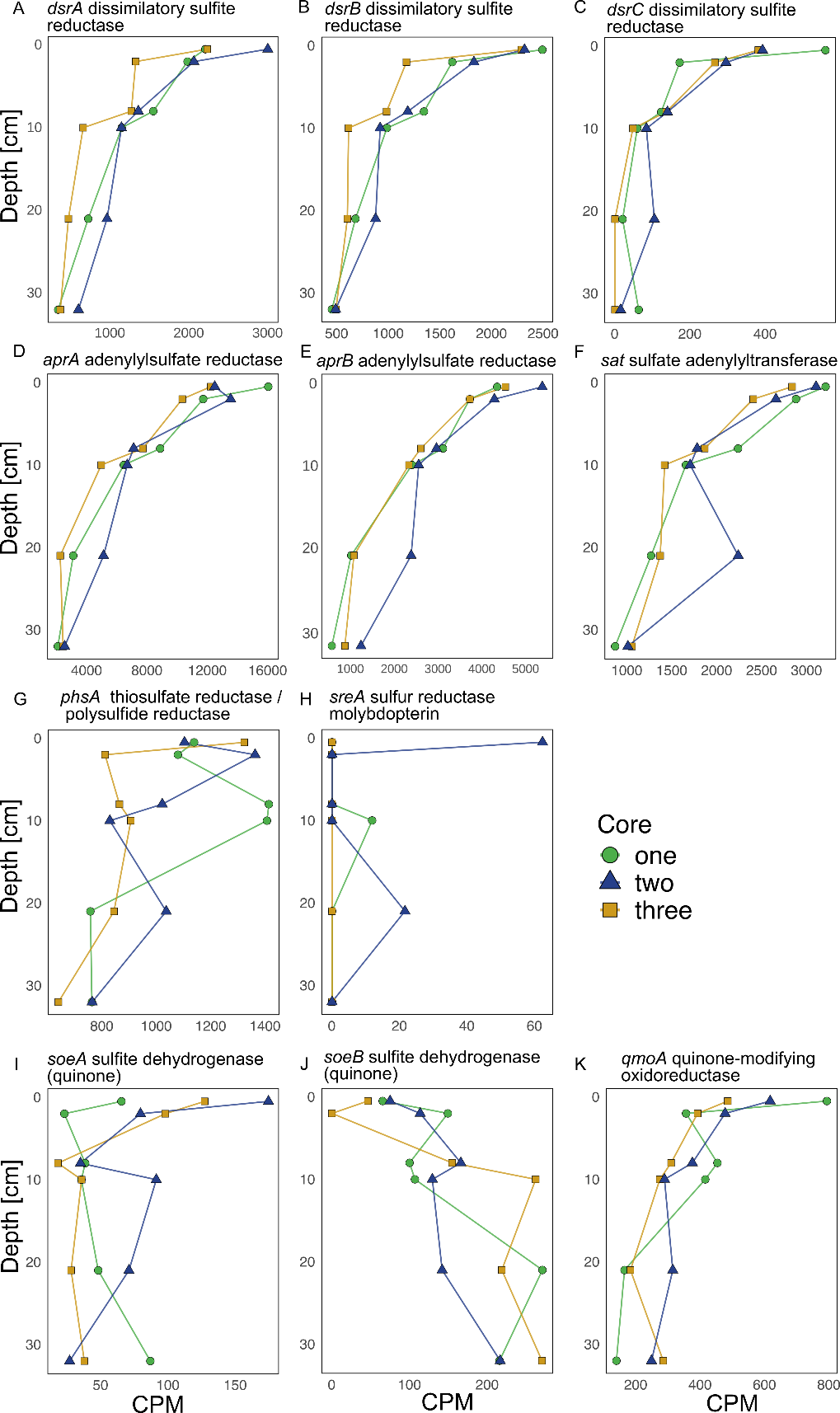


Figure S13 | Gene expression profiles linked to sulphur in Köljo Fjord. Gene expression profiles by depth of genes associated with sulphur metabolism.


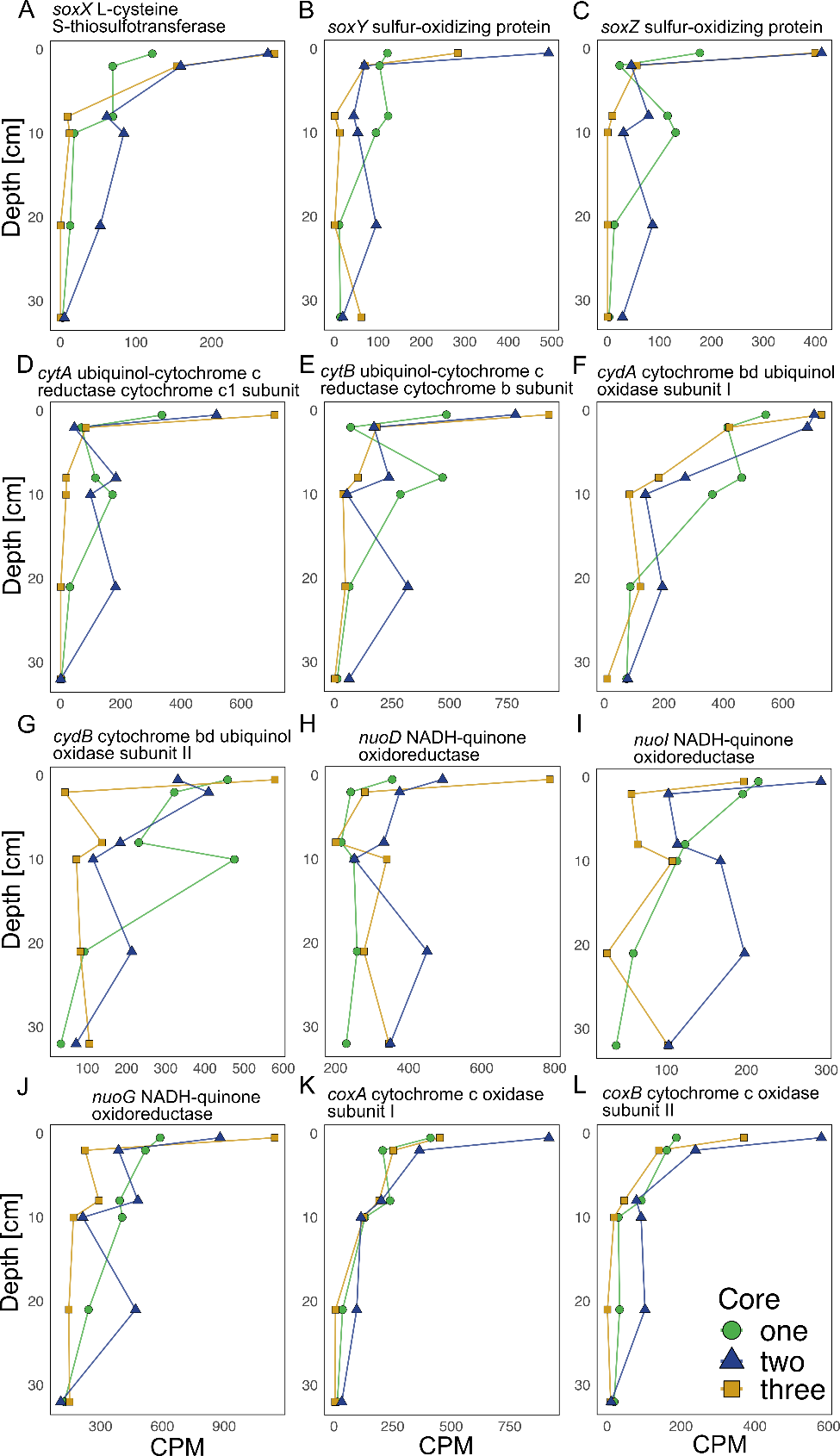


Figure S14 | Gene expression profiles linked to sulphur and electron transfer in Köljo Fjord. A-C) Gene expression profiles by depth of genes associated with sulphur metabolism and D-L) electron transfer

Table S1 | Characteristics of the stations studied. Stations on the west coast of Sweden (SE) and the Tvärminne archipelago in southern Finland (FI). BW = Bottom water.

| **Station** | **Water depth [m]** | **Latitude** | **Longitude** | **BW salinity** | **BW O_2_ [mg/L]** | **BW redox conditions** |
| --- | --- | --- | --- | --- | --- | --- |
| Koljö Fjord (SE) | 12 | 58°15'28" | 11°34'5" | 32.7 | 1.4 | hypoxic |
| Kristineberg Bay (SE) | 40 | 58°14'58" | 11°26'32" | 24.0 | 4.4 | oxic |
| Nearshore (FI)* | 13.5 | 59°55'22" | 23°20'18" | 5.9 | 4.2 | oxic |
| Offshore (FI)* | 22.5 | 59°51'28" | 23°22'31" | 6.2 | 1.7 | hypoxic |

*Sampling and data reported by Hermans *et al*. (2024)

Table S2 | Kruskal-Wallis test of *Candidatus* Electrothrix abundance in Offshore Tvärminne station samples. Chi-squared = 26.385, df = 9, p-value = 0.001767. Duncan’s test on Kruskal-Wallis multiple comparison p-values adjusted with the Benjamini-Hochberg method.

| **Depth Comparison [cm]** | | **Z** | **P.unadj** | **P.adj** |
| --- | --- | --- | --- | --- |
| 0-1 | 1-3 | -0.13912167 | 0.889354006 | 0.9095666 |
| 0-1 | 11-13 | 2.13319892 | 0.032908412 | 0.13462532 |
| 1-3 | 11-13 | 2.27232059 | 0.023067154 | 0.12975274 |
| 0-1 | 20-22 | 3.01430282 | 0.002575706 | **0.02318135** |
| 1-3 | 20-22 | 3.15342449 | 0.00161367 | **0.02420505** |
| 11-13 | 20-22 | 0.8811039 | 0.378261586 | 0.50064033 |
| 0-1 | 3-5 | 0.83473001 | 0.403869739 | 0.51926109 |
| 1-3 | 3-5 | 0.97385168 | 0.330130188 | 0.47922124 |
| 11-13 | 3-5 | -1.29846891 | 0.194126254 | 0.41598483 |
| 20-22 | 3-5 | -2.17957281 | 0.029289142 | 0.13180114 |
| 0-1 | 30-31 | 2.92155504 | 0.003482887 | **0.02612165** |
| 1-3 | 30-31 | 3.06067671 | 0.002208374 | **0.02484421** |
| 11-13 | 30-31 | 0.78835612 | 0.430488428 | 0.53811054 |
| 20-22 | 30-31 | -0.09274778 | 0.926103938 | 0.92610394 |
| 3-5 | 30-31 | 2.08682503 | 0.036903954 | 0.13838983 |
| 0-1 | 40-42 | 3.15342449 | 0.00161367 | **0.03630757** |
| 1-3 | 40-42 | 3.29254616 | 0.000992846 | **0.04467807** |
| 11-13 | 40-42 | 1.02022557 | 0.307621493 | 0.4773437 |
| 20-22 | 40-42 | 0.13912167 | 0.889354006 | 0.93071931 |
| 3-5 | 40-42 | 2.31869448 | 0.020411608 | 0.13121748 |
| 30-31 | 40-42 | 0.23186945 | 0.816639415 | 0.89631155 |
| 0-1 | 5-7 | 1.25209502 | 0.210535243 | 0.41191678 |
| 1-3 | 5-7 | 1.39121669 | 0.164159728 | 0.36935939 |
| 11-13 | 5-7 | -0.8811039 | 0.378261586 | 0.51581125 |
| 20-22 | 5-7 | -1.7622078 | 0.078034194 | 0.19508549 |
| 3-5 | 5-7 | 0.41736501 | 0.676411447 | 0.80101356 |
| 30-31 | 5-7 | -1.66946002 | 0.095026246 | 0.22506216 |
| 40-42 | 5-7 | -1.90132947 | 0.057258871 | 0.16104057 |
| 0-1 | 7-9 | 0.97385168 | 0.330130188 | 0.49519528 |
| 1-3 | 7-9 | 1.11297335 | 0.265719875 | 0.45989978 |
| 11-13 | 7-9 | -1.15934724 | 0.246314673 | 0.46184001 |
| 20-22 | 7-9 | -2.04045114 | 0.041305413 | 0.14298027 |
| 3-5 | 7-9 | 0.13912167 | 0.889354006 | 0.95287929 |
| 30-31 | 7-9 | -1.94770336 | 0.051450468 | 0.1543514 |
| 40-42 | 7-9 | -2.17957281 | 0.029289142 | 0.14644571 |
| 5-7 | 7-9 | -0.27824334 | 0.780825569 | 0.87842877 |
| 0-1 | 9-11 | 1.85495558 | 0.06360257 | 0.16835974 |
| 1-3 | 9-11 | 1.99407725 | 0.046143613 | 0.14831876 |
| 11-13 | 9-11 | -0.27824334 | 0.780825569 | 0.90095258 |
| 20-22 | 9-11 | -1.15934724 | 0.246314673 | 0.44336641 |
| 3-5 | 9-11 | 1.02022557 | 0.307621493 | 0.49439169 |
| 30-31 | 9-11 | -1.06659946 | 0.286152744 | 0.47692124 |
| 40-42 | 9-11 | -1.29846891 | 0.194126254 | 0.39707643 |
| 5-7 | 9-11 | 0.60286056 | 0.546601452 | 0.66478555 |
| 7-9 | 9-11 | 0.8811039 | 0.378261586 | 0.53193035 |

Table S3 | **Results of the Kruskal-Wallis test performed on the abundance of *Candidatus* Electrotrhix, based on the 13 partial 16S rRNA gene sequences identified in samples from Koljö Fjord stratified by depth. Duncan's test was conducted on Kruskal-Wallis multiple comparison p-values, which were adjusted using the Benjamini-Hochberg method.**

| **Depth** | **Comparison [cm]** | **Z** | **P.unadj** | **P.adj** |  |
| --- | --- | --- | --- | --- | --- |
| 0-1 | 1-3 | -1.5802906 | 0.1140403312 | 0.285100828 |  |
| 0-1 | 20-22 | 0.837068 | 0.4025543276 | 0.503192909 | |
| 1-3 | 20-22 | 2.4173586 | **0.0156336053** | **0.078168026** |  |
| 0-1 | 30-32 | 1.9523208 | **0.0509001249** | 0.152700375 |  |
| 1-3 | 30-32 | 3.5326114 | **0.0004114768** | **0.006172151** |  |
| 20-22 | 30-32 | 1.1152528 | 0.2647421033 | 0.397113155 |  |
| 0-1 | 7-9 | -0.6845692 | 0.4936158383 | 0.569556736 |  |
| 1-3 | 7-9 | 0.8957214 | 0.3704015517 | 0.505093025 |  |
| 20-22 | 7-9 | -1.5216372 | 0.1281000146 | 0.274500031 |  |
| 30-32 | 7-9 | -2.63689 | **0.0083669961** | **0.062752471** |  |
| 0-1 | 9-11 | 0.5865342 | 0.5575166144 | 0.59733923 |  |
| 1-3 | 9-11 | 2.1668248 | **0.0302482188** | 0.11343082 |  |
| 20-22 | 9-11 | -0.2505339 | 0.8021745106 | 0.802174511 |  |
| 30-32 | 9-11 | -1.3657867 | 0.172005928 | 0.322511115 |  |
| 7-9 | 9-11 | 1.2711033 | 0.2036918979 | 0.339486497 |  |

**References**

Algeo, T.J., Li, C., 2020. Redox classification and calibration of redox thresholds in sedimentary systems. Geochimica et Cosmochimica Acta 287, 8–26. <https://doi.org/10.1016/j.gca.2020.01.055>

Paul, K.M., Hermans, M., Jokinen, S.A., Brinkmann, I., Filipsson, H.L., Jilbert, T., 2023. Revisiting the applicability and constraints of molybdenum and uranium-based paleo redox proxies: comparing two contrasting sill fjords (preprint). Biogeochemistry: Coastal Ocean. https://doi.org/10.5194/bg-2023-83

Bushmanova, E., Antipov, D., Lapidus, A., Prjibelski, A.D., 2019. rnaSPAdes: a *de novo* transcriptome assembler and its application to RNA-Seq data. GigaScience 8, giz100. <https://doi.org/10.1093/gigascience/giz100>

Chen, Y.-L., Lee, C.-C., Lin, Y.-L., Yin, K.-M., Ho, C.-L., Liu, T., 2015. Obtaining long 16S rDNA sequences using multiple primers and its application on dioxin-containing samples. BMC Bioinformatics 16, S13. https://doi.org/10.1186/1471-2105-16-S18-S13

Aroney, S.T.N., Newell, R.J.P., Nissen, J.N., Camargo, A.P., Tyson, G.W., Woodcroft, B.J., 2025. CoverM: read alignment statistics for metagenomics. Bioinformatics 41, btaf147. https://doi.org/10.1093/bioinformatics/btaf147

Hermans, M., Stranne, C., Broman, E., Sokolov, A., Roth, F., Nascimento, F.J.A., Mörth, C.-M., Ten Hietbrink, S., Sun, X., Gustafsson, E., Gustafsson, B.G., Norkko, A., Jilbert, T., Humborg, C., 2024. Ebullition dominates methane emissions in stratified coastal waters. Science of The Total Environment 945, 174183. https://doi.org/10.1016/j.scitotenv.2024.174183
